# Mrc1 and Tof1 prevent fragility and instability at long CAG repeats by their fork stabilizing function

**DOI:** 10.1093/nar/gky1195

**Published:** 2018-11-23

**Authors:** Lionel Gellon, Simran Kaushal, Jorge Cebrián, Mayurika Lahiri, Sergei M Mirkin, Catherine H Freudenreich

**Affiliations:** Department of Biology, Tufts University, Suite 4700, 200 Boston Ave, Medford, MA 02155, USA

## Abstract

Fork stabilization at DNA impediments is key to maintaining replication fork integrity and preventing chromosome breaks. Mrc1 and Tof1 are two known stabilizers that travel with the replication fork. In addition to a structural role, Mrc1 has a DNA damage checkpoint function. Using a yeast model system, we analyzed the role of Mrc1 and Tof1 at expanded CAG repeats of medium and long lengths, which are known to stall replication forks and cause trinucleotide expansion diseases such as Huntington's disease and myotonic dystrophy. We demonstrate that the fork stabilizer but not the checkpoint activation function of Mrc1 is key for preventing DNA breakage and death of cells containing expanded CAG tracts. In contrast, both Mrc1 functions are important in preventing repeat length instability. Mrc1 has a general fork protector role that is evident at forks traversing both repetitive and non-repetitive DNA, though it becomes crucial at long CAG repeat lengths. In contrast, the role of Tof1 in preventing fork breakage is specific to long CAG tracts of 85 or more repeats. Our results indicate that long CAG repeats have a particular need for Tof1 and highlight the importance of fork stabilizers in maintaining fork integrity during replication of structure-forming repeats.

## INTRODUCTION

DNA replication is a robust process that allows the transmission of the genetic information to a daughter cell with a high level of fidelity. However, replication faces numerous impediments that perturb its progression and can lead to a replication fork stall. These impediments can be a tightly bound protein, damaged or cross-linked nucleotides, or DNA structures (1–3). In order to achieve fork restart and completion of replication, the stalled fork needs to be stabilized. Failure to do so will lead to extensive generation of ssDNA due to the uncoupling of the polymerase and the helicase, increasing the likelihood of fork collapse and chromosome breakage. Replicative stress is the hallmark of cells with activated oncogenes, and is one cause of the genome instability that occurs in early stages of tumorigenesis (4–7).

Inverted repeats, hairpin-forming repeats, and triplex structures have all been shown to stall replication forks in bacterial, yeast, and mammalian cells (8–15). Contrary to natural protein impediments that have emerged to protect the genome and fork blocking DNA damage that has evolved associated DNA repair mechanisms, DNA structures cannot easily be bypassed and represent a particular threat for genome integrity.

Expanded CAG repeats are responsible for several inherited neurodegenerative diseases including Huntington's disease, myotonic dystrophy type 1 and several types of spinocerebellar ataxia (16,17). CAG/CTG (CAG) repeats form hairpins *in vitro* (18–21) and *in vivo* (22). Hairpin formation during replication or repair can lead to repeat length changes referred to as repeat instability, including expansions and contractions (reviewed in (16)). Compared to expanded CGG or GAA repeats, long CAG repeats are a weaker barrier for fork progression as observed by 2D gel analysis in *Saccharomyces cerevisiae* (23–26) or by a quantitative PCR-based nascent DNA abundance assay in human cells (27). However, expanded CAG repeats appear to be especially prone to fork reversal, as visualized by two dimensional (2D) gel electrophoresis and electron microscopy (EM) (24,26,28). For a CAG tract of about 100 repeats, up to 30% of replication intermediates traversing expanded CAG tracts are converted into joint molecules (26). Thus, studying how the replication fork traverses expanded CAG tracts is of great interest for understanding their genetic instability.

It has been shown that CAG tracts of 45 units or longer can induce length-dependent breakage of a yeast chromosome, referred to as fragility, as measured by a sensitive genetic assay (29,30). For longer (CAG)_250_, (CGG)_160_ and (GAA)_230_ repeats, DNA breakage was directly visualized by Southern blot (29,31–33). Thus, fragility represents a hallmark of expanded TNRs and can be used to reveal factors that participate in fork stability, since unprotected replication forks are prone to collapse and breakage.

Mrc1 (hClaspin), Tof1 (hTimeless) and Csm3 (hTipin) are three proteins that associate with the replication fork via physical interaction with components of the MCM helicase, Cdc45 and the replicative DNA polymerases. Associations between Mrc1/Claspin, Polϵ and MCM subunits have been revealed by co-immunoprecipitation and yeast two hybrid experiments (34–37). A fluorescence co-localization assay along with pull-down experiments were used to reveal direct interactions between Tof1/Timeless, and Mcm2-7 as well as Polα/δ/ϵ (34,38,39). Similarly, interactions between Csm3/Tipin and Mcm7, Polα, Ctf4, and RPA have been described (34,40–42). These interactions allow Mrc1, Tof1 and Csm3 to travel with the fork and facilitate a normal speed of DNA synthesis (43–46). In *S. cerevisiae*, Tof1 forms a stable complex with Csm3 that binds to the chromatin and to Mrc1 (47,48).

In addition to the DNA synthesis rate function, Mrc1-Tof1-Csm3 have an important role in fork protection. The absence of Mrc1 or Tof1-Csm3 causes the uncoupling of Polϵ from the MCM helicase, leading to extensive DNA unwinding and ssDNA formation at forks stalled by HU (35,43,49). It was suggested that by tethering the MCM helicase to the polymerase, Mrc1 and Tof1-Csm3 can restrain the progression of the helicase when DNA synthesis is inhibited and prevent the fork from becoming uncoupled. The complex may also prevent accumulation of ssDNA by restraining Exo1-mediated degradation of nascent strands at stalled forks (50). Experiments testing replication through inverted repeats or a (CGG)_40_ tract revealed increased fork stalling in *tof1Δ* and *mrc1Δ* yeast cells, suggesting that they are both required for fork progression through some DNA structure barriers (9,10). Consistently, absence of Mrc1 or Tof1 results in a significant increase in gross chromosomal rearrangements at an expanded GAA repeat tract (33). In contrast, deletion of *TOF1* reduced repeat fragility at an expanded ATTCT repeat that is a DNA unwinding element but does not form a stable DNA structure (51), and fork stalling is reduced at protein-mediated barriers in *tof1Δ* cells (52). Therefore, the role of Tof1 is dependent on the type of fork perturbation. The role of Mrc1 and Tof1 on replication through a long CAG tract has not been previously studied.

The Mrc1–Tof1–Csm3 complex also has a role in the checkpoint response to DNA damage, a function that requires phosphorylation of multiple SQ motifs in the Mrc1 protein (10,53,54). Notably, Mrc1 is required for Rad53 activation and cell cycle arrest in response to replication stress (53,55–58). In a previous study, we demonstrated the involvement of several key checkpoint proteins (Rad9/53BP1, Mec1/ATR, Rad17-Rad24 (9–1–1 clamp/clamp loader) and Rad53/Chk2) in preventing CAG fragility and instability (mainly contractions) of medium (85) and long (135–155) CAG repeats (59). Expansions, contractions and fragility of a medium and long CAG/CTG tract were also elevated in strains with the *mrc1-1* checkpoint-deficient allele (60), however it was not determined if there were additional Mrc1 defects in addition to a defective replication checkpoint responsible for these phenotypes, and the role of Tof1 was not tested. Rad53, Tof1 and Mrc1 were identified as important for preventing CAG repeat instability of short (CAG)_13–20_ repeats, which are below the length known to stall replication (61). In human cells, knockdown of Claspin, Timeless, or Tipin all significantly increased expansions and contractions of expanded CAG tracts of 100 or more repeats, confirming the importance of this complex in preventing instability of longer CAG repeat tracts in mammals (27). These experiments illustrate the important role of the Mrc1–Tof1–Csm3/Claspin-Timeless–Tipin complex in preventing CAG instability, but the role of its fork stabilizing versus its checkpoint function on stability of long expanded repeat tracts was not determined. In addition, its function in fork stalling and recovery at CAG/CTG DNA structures was not clear.

Given the importance of the Mrc1/Tof1/Csm3 complex in fork stabilization, repeat stability, and its potentially special role at forks stalled by DNA structures, it was important to investigate its function at expanded CAG repeats of lengths that detectably stall forks and cause chromosome fragility. Therefore, we investigated the role of the Mrc1 and Tof1 proteins on the fragility, instability, and replication of medium (70–85) and long (135–155) expanded CAG repeats integrated into an artificial chromosome in *S. cerevisiae*. Our results show that both proteins are required to prevent chromosome fragility, to maintain the length integrity of the repeats, and to facilitate replication through long repeats that constitute a visible fork barrier. However, significant differences between the proteins were identified: Mrc1 is required to prevent breaks at all repeat lengths as well as at non-repetitive DNA, whereas Tof1 has a specific role in preventing fragility of long CAG repeats that stall replication forks. We further demonstrate that the role of Mrc1 in protecting against CAG fragility mainly involves its fork protection function. In contrast, the prevention of repeat instability requires both functions of Mrc1.

## MATERIALS AND METHODS

### Yeast strains

Yeast strains used in this study are listed in Supplementary Table S4. The triplet repeat sequences reported here all have the CAG repeat on the lagging strand template of YAC CF1, and CTG repeats on the Okazaki fragment. This CAG nomenclature is used throughout. The medium tract size is CAG-70 for BY4705 background strains and CAG-85 for W303 background strains. Long tract size varies from CAG-135 to CAG-155 (see Supplementary Table S4 for strain details). Deletion mutants were created using one-step gene replacement (62,63) in WT, BY4705 or W303 backgrounds containing either medium, long or no CAG tracts. Gene disruptions were confirmed by PCR for absence of the open reading frame and presence of both junctions. For the *mrc1^AQ^* strains, obtained from the Pasero lab, YACs carrying the CAG-85, or CAG-155 tract and a control YAC without a CAG tract (CAG-0) were introduced by cytoduction (64). CAG repeat length from a portion of the colony was determined by colony PCR (30). Starting colonies with intact tract lengths were chosen for experiments. All experiments were performed at 30°C with at least two independent transformants or cytoductants.

### CAG fragility and instability assays

Fragility assays were performed as in (30) and described in (65). A single starting colony with correct tract length was suspended in 1 ml sterile H2O and used to inoculate 10 separate YC-Leu cultures that were grown for 6–7 doublings at 30°C to maintain selection for the YAC, but allow loss of the right arm. 100 μl of each culture was plated on FOA-Leu to select for breakage events and a portion of each culture was combined and plated for single colonies on YC-Leu for a total cell count. Mutation rate was determined using the method of maximum likelihood (66) and data presented are an average of 3–8 experiments (Supplementary Table S1). Error bars indicate the standard error of the mean. Significance compared to the WT value for the same tract length was determined using a pooled variance*t*-test. Contraction and expansion frequencies for medium CAG-70 to CAG-85 and long CAG-135 to CAG-155 tracts were determined as described previously (30). For each strain, 155–335 colonies were analyzed for CAG repeat length by colony PCR in at least three separate experiments, using primers flanking the CAG repeat. PCR products were separated on a 2% Metaphor gel (Cambrex Bio Science Rockland, Inc.) and sized. The frequency of repeat expansions and contractions in each strain background was calculated and statistical significance determined by the Fisher's Exact test (Supplementary Table S2).

### Analysis of replication intermediates by 2D gels

Seventy or 130 CAG repeats were cloned between *Hind*III and *EcoR*I of a pYES2 plasmid. Plasmids were transformed into WT, *tof1*Δ or *mrc1*Δ, yeast (gift from H. Klein lab) by the lithium acetate method (67) and selection on media lacking uracil. Cells were grown at 30°C in YC-Ura (synthetic medium without uracil) until OD_600_ of 1.5. The length of repeats tracts were confirmed before and after the cell culture by yeast colony PCR as described in (65) with primers flanking the inserts, and amplicons were resolved on a 2% Metaphor gel.

Replication intermediates were isolated according to the ‘QIAGEN genomic DNA Handbook,’ using genomic-tip 100/G columns. DNA was digested by *Nde*I, *BciV*I and *Psi*I (New England Biolabs) for 7 h at 37°C. First-dimension gels (0.4% agarose in 1× TBE) were run at 1 V/cm for 22 h at room temperature, while second-dimension gels (1% agarose in 1× TBE) were run at 5 V/cm for 9 h at 4°C in the presence of 0.3 μg/ml ethidium bromide. Gels were washed 15 min in 0.25 N HCl before an overnight transfer to a charged nylon membrane (Hybond-XL, GE Healthcare) in 0.4 N NaOH. Hybridization was performed overnight with a 413 bp randomly primed probe, corresponding to the Gal1 promoter of pYES2 plasmid. Membranes were washed twice with washing solution I (SSC 2×, 1% SDS) pre-heated at 65°C and twice with washing solution II (SSC 0.1×, 0.1% SDS) pre-heated at 42°C. Membranes were exposed on IR-sensitive screens for 1–5 days and detection was performed on a Pharos FX PhosphorImager (Bio-Rad). Densitograms were done with NIH ImageJ and quantification analysis was done as described in (8). Statistical analysis was performed with GraphPad Prism software. Mean percentage of replication slowing values was compared by two-way ANOVA followed by the Fisher's LSD test.

### Microcolony analysis

Actively dividing cells from a colony with the correct CAG tract length verified by PCR were transferred onto yeast complete solid media lacking Leucine (YC-Leu). Single unbudded normal-sized G_1_ cells were micromanipulated to designated locations on the plate using a Nikon Eclipse E400 or a Singer MSM400 tetrad dissection scope and allowed to divide for 30 h at 30°C. Pictures were taken at 10× magnification using an Olympus microscope, and microcolony area for 30 h was measured using NIH ImageJ software. Based on pilot survival experiments to discriminate dividing versus non-dividing microcolonies, survivors were defined as area ≥0.016 mm^2^ and non-survivors were defined as area <0.016 mm^2^ for 30 h. Differences between repeat lengths for a particular genotype were determined by ANOVA and Fisher's LSD statistical analysis. To generate a graphical illustration of the different size distributions, the survivor areas were binned into sections of 0.033 mm^2^ increments (starting at 0.016 mm^2^) and graphed using the Prism curve-fitting software (GraphPad Software, San Diego, CA, USA). For the percentage of non-survivors, statistical significance between tract lengths was determined by a Fisher's exact test. The microcolony data and statistical analysis are presented in Supplementary Table S3.

## RESULTS

### Mrc1 and Tof1 are required for prevention of CAG repeat fragility with a specific function for Tof1 at long CAG tracts

To explore the role of the Mrc1/Tof1/Csm3 fork stabilization complex in the maintenance of CAG repeats, we performed assays for CAG fragility and instability in yeast cells deleted for the *MRC1* or *TOF1* genes. CAG fragility was measured by a genetic assay that detects chromosome end-loss resulting from chromosome breakage at or near the repeat tract; a telomere seed sequence proximal to the repeat facilitates recovery of broken chromosomes, which results in loss of the distal *URA3* gene and thus 5-FOA resistant colonies (Supplementary Figure S1). The assay captures only a fraction of breakage events, those that are not able to heal normally but rather result in chromosome end loss, however it is useful for making comparisons between repeat sizes or between WT and mutant strains. The fragility assay was performed for WT and mutant strains in the absence of a CAG repeat (no tract), or in the presence of a medium (70–85) or long (135–155) CAG tract, lengths in the range known to cause replication perturbation and fragility in wild-type cells.

**Figure 1.**
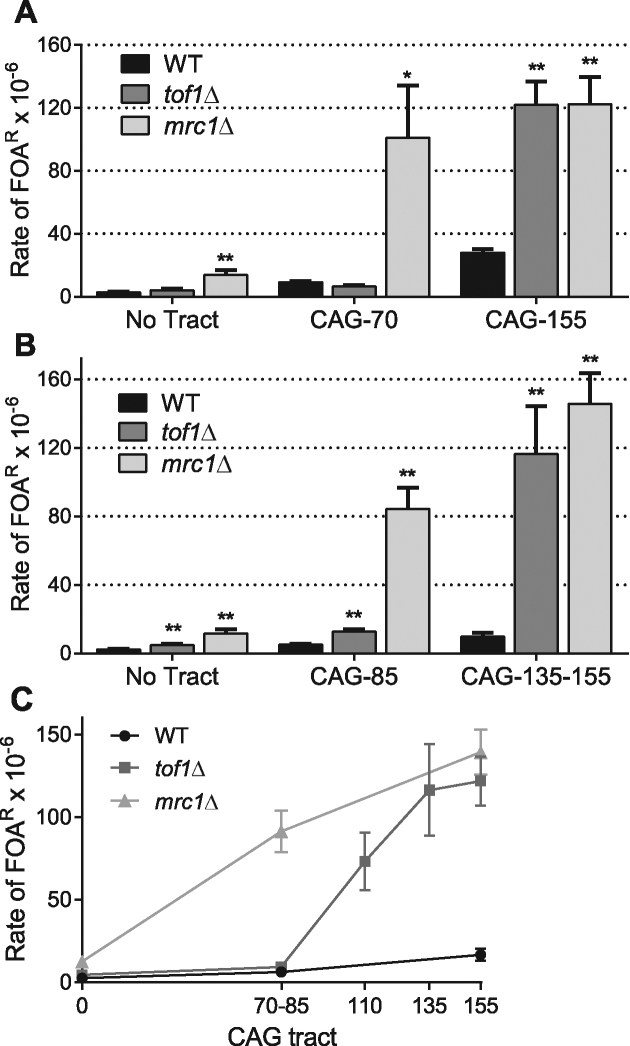
Tof1 and Mrc1 are required to prevent chromosomal breakage of DNA sequences containing expanded CAG repeats. (**A**) Fragility assays (Figure S1) were performed on WT, *tof1*Δ and *mrc1*Δ strains (W303 background) harboring a YAC with CAG-70 repeats, CAG-155 repeats or no tract; data presented are an average of 3–7 experiments (see Supplementary Table S1). Mutation rate was determined using the method of maximum likelihood. Error bars indicate the standard error of the mean (SEM). Significance compared to the WT value for the same tract length was determined using a pooled variance *t*-test, *P < 0.05; **P < 0.01. (**B**) As in (A) except the strains background is BY4705 and CAG-85 was used as a medium tract, and CAG-135 (*tof1*Δ) or CAG-155 (WT, *mrc1*Δ) as a long tract. (**C**) The rate of FOA^R^ for each tract length tested in the indicated strains. When values for a particular tract length were available from both BY and W303 strain backgrounds, they were averaged. Significance to WT with the same tract length and genotype determined as in (A). Exact values and numbers analyzed are in Supplementary Table S1.

In the absence of the full Mrc1 protein, we observed a significant increase in the rate of FOA resistance at all tract lengths tested (Figure 1A and B). The fragility rate measured in two yeast backgrounds (BY4705 and W303) shows a consistent result: breaks were increased in *mrc1*Δ cells compared to WT cells 5-fold without a repeat, 11- to 17-fold for the medium CAG tracts, and 4- to 15-fold for the long tract (Supplementary Table S1). Although a significant increase in chromosome fragility is evident in the absence of CAG repeats, the presence of expanded CAG repeats dramatically increases the number of breakage events recovered in the *mrc1*Δ background.

The absence of the *TOF1* gene revealed a different outcome. Whereas no fragility phenotype was observed for the no tract or CAG-70 tract in *tof1*Δ cells compared to the WT BY4705 strain, the presence of 155 CAG repeats in the *tof1*Δ cells significantly raised the fragility rate. To validate this phenotype, we repeated the assay in a different strain background (W303) with CAG-85 and CAG-135 repeats. Fragility of the CAG-135 tract was significantly increased over WT by 12-fold (Figure 1B). The CAG-85 medium tract showed a lesser but still significant 2.5-fold increase in fragility in *tof1*Δ cells compared to WT. This data suggests that Tof1 has a role in preventing CAG tract breakage that is highly specific to the number of CAG repeats, and in the presence of long repeats Tof1 is as important as Mrc1. To further investigate the *tof1Δ* fragility profile, we performed the assay with a CAG-110 tract. At this length, the strain shows a fragility rate significantly above the rate obtained for CAG-70 or CAG-85, albeit less pronounced than the CAG-135 or -155 tract rates (Figure 1C). This data defines a threshold of around 85 CAG repeats where Tof1 becomes important for preventing fragility.

### Both Mrc1 and Tof1 are required for prevention of CAG repeat contractions, but Mrc1 has a more vital role in preventing expansions

To determine the contribution of the Mrc1–Tof1–Csm3 complex in prevention of repeat instability, contractions and expansions were determined in the various mutant conditions by a sensitive PCR assay (65). Both expansions and contractions were dramatically increased in the *mrc1*Δ mutant for both the medium and the long CAG tracts (Table 1, Supplementary Table S2). Notably, 67% of the *mrc1*Δ cells acquired a contraction at the long tract length during the 6–8 cell divisions of growth utilized for the instability assay, compared to 18% for WT. Despite the high contraction frequency, a 7-fold increase in expansion frequency over the WT was also observed. In total, ¾ of the *mrc1*Δ cells underwent an instability event at the long CAG tract during the course of the experiment. Thus, the Mrc1 protein is extremely important for preserving integrity of medium and long CAG repeat tracts.

**Table 1.** CAG instability data

		Contractions	Expansions	
Genotype	CAG repeat length	% (fold over WT)	% (fold over WT)	Total # reactions
WT	85	6.0	1.1	184
*tof1*Δ	85	26** (4.4)	1.4 (1.3)	207
*mrc1*Δ	85	24** (4.0)	6.0** (5.5)	335
*mrc1AQ*	85	11 (1.8)	2.8 (2.5)	319
*mrc1-1*	85	10 (1.7)	5.8* (5.3)	156
*rad53-21*	85	21** (3.4)	4.4 (4.0)	156
WT	135	18	1.3	155
*tof1*Δ	135	69** (3.8)	1.5 (1.2)	197
*mrc1*Δ	155	67** (3.7)	7.0** (5.4)	319
*mrc1AQ*	145	43** (2.4)	3.0 (2.3)	299
*mrc1-1*	155	30* (1.7)	1.3 (1.0)	156
*rad53-21*	135	37** (2.1)	1.9 (1.5)	155

*P ≤ 0.05, ** *P* ≤ 0.01 compared to wild-type of the same tract length, using Fisher's exact test; see also Supplementary Table S2.

Interestingly no significant increase in the number of expansions were detected for the*tof1*Δ mutant, however contractions were as high as the frequency observed for the *mrc1*Δ mutant for both the medium and long CAG tracts with 26% or 69% of repeats contracted for CAG-85 or 135, respectively (Table 1, Supplementary Table S2). Thus, Tof1 also has an important role in maintaining CAG stability, specifically in preventing contractions.

### Tof1 and Mrc1 facilitate replication through expanded CAG-130 tracts

**Figure 2.**
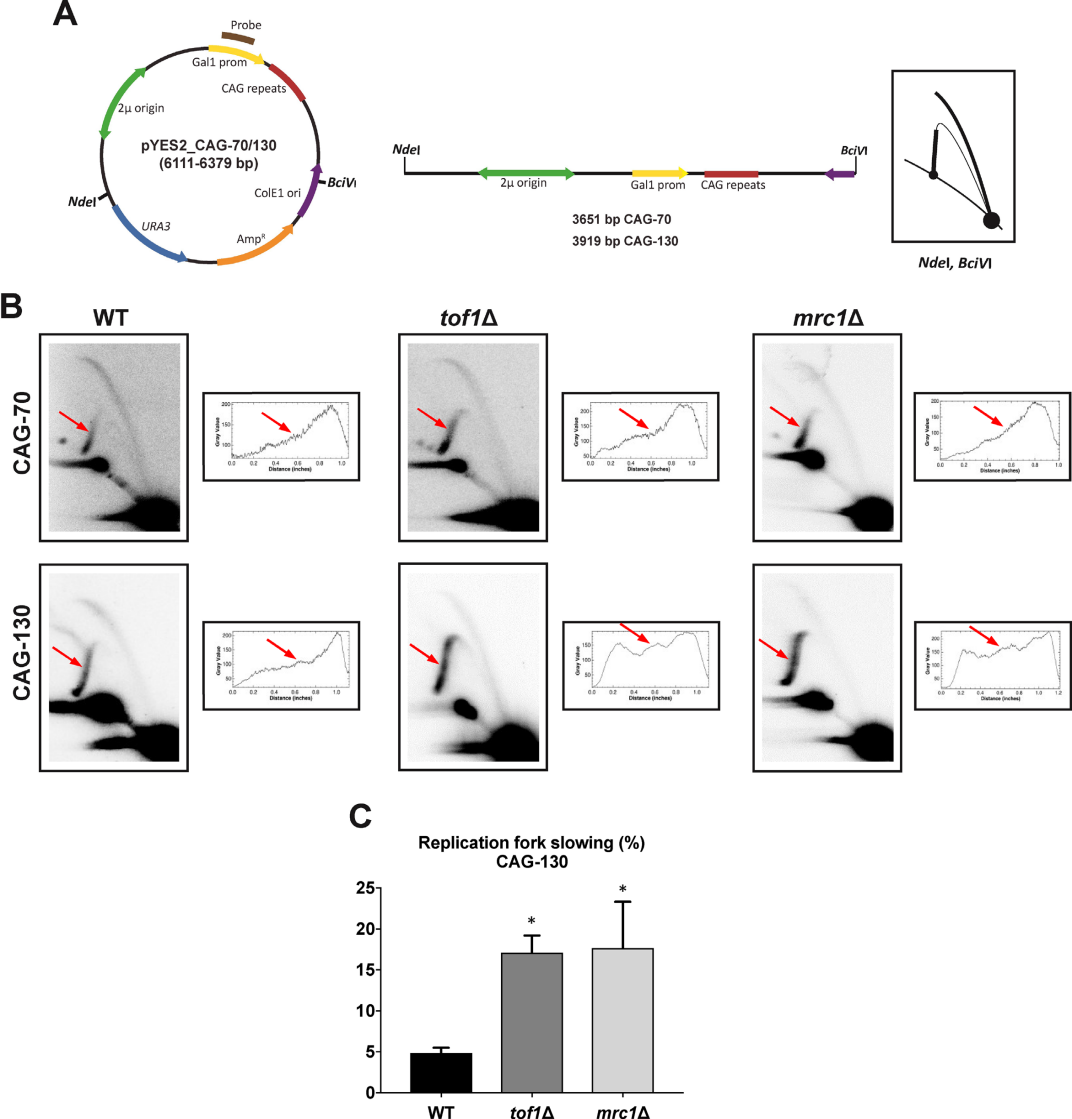
Analysis of replication through CAG-70 and CAG-130 repeats by two-dimensional (2D) agarose gel electrophoresis in WT, *tof1*Δ and *mrc1*Δ strains. (**A**) Schematic of the pYES2 constructs is shown with its mass and genetic map. The relative positions of its most relevant features are indicated inside: the 2 μm origin, the ColE1 unidirectional origin (ColE1 Ori), the ampicillin-resistance gene (Amp^R^), *URA3*, the *GAL1* promoter (Gal1 prom) and 70 or 130 CAG repeats. Outside, the relative positions of sites recognized by the restriction endonucleases *Nde*I and *BciV*I are indicated. To the right, is shown the corresponding linear map of the pYES2 plasmid restriction fragment with the sizes and the diagrammatic interpretation if replication initiates bi-directionally at the 2 μm origin and proceeds unconstrained. (**B**) Representative 2D gels of replication through CAG-70 and CAG-130 repeats in WT, *tof1*Δ, and *mrc1*Δ strains. DNA was isolated, digested with *Nde*I and *BciV*I and analyzed by 2D gel. Red arrow points to the location of the CAG repeats. To the right of each 2D gel are shown the densitometric profiles corresponding to the Y-arc region where the (CAG)n repeats are located; peaks on densitograms correspond to bulges on the Y-arcs. A representative gel and its corresponding profile is shown; three experiments were analyzed for each strain. (**C**) Quantification of replication fork slowing in pYES2 CAG-130 in WT, *tof1*Δ and *mrc1*Δ strains. The ratio of radioactivity in the peak area to that corresponding area of a smooth replication arc reflects the extent of replication slowing. Three different experiments were performed for each strain. Percentage of replication fork slowing is 3.3%, 4.6% and 5.8% for WT, 13.2%, 17.7% and 20.4% for *tof1*Δ, and 8.3%, 16.4% and 27.7% for *mrc1*Δ. Error bars indicate standard error of the mean. The star indicates a significant difference between wild-type and mutants. *P* = 0.0483 (*tof1*Δ versus WT), *P* = 0.0378 (*mrc1*Δ versus WT).

Considering the unique requirement for Tof1 to prevent breakage of long CAG tracts of greater than 85 repeats, we wanted to determine the replication profile through the CAG tract in cells lacking Tof1 or Mrc1 in comparison to wild-type. CAG-70 or CAG-130 tracts were cloned into a yeast replicating plasmid and replication intermediates were isolated and separated by size and shape on a 2D gel (Figure 2). Since expanded CAG tracts were previously shown to give a distinct yet weak stall when placed on a yeast chromosome (25,26), digests were chosen to place the potential stall site on either the descending (Figure 2) or ascending (Supplementary Figure S2) arm of the arc of replication intermediates. At CAG-70, no replication fork stall or pausing was visible at the expected location in any of the strain backgrounds (Figure 2B). However, for CAG-130 a weak but distinct pausing site was visible at the site of the repeat in WT cells, which was further increased in both *tof1*Δ and *mrc1*Δ mutants. Quantification of the difference from three independent experiments showed a significant 3.7-fold increase in both mutants compared to WT. A similar trend was observed when the digest was performed so that the CAG tract was on the ascending arm, where pausing can lead to double Y structures from converging forks (joint molecules, Supplementary Figure S2). Note that in this plasmid system, unlike on a yeast chromosome, repeat-dependent reversed fork structures were not observed, which could be due to the different topology, differences in chromatin structure, or the quick convergence of the incoming fork. Therefore, Mrc1 and Tof1 proteins play an important role in facilitating replication through long CAG tracts which correlates with the increased fragility of these tracts in cells lacking Mrc1 or Tof1. We conclude that Tof1 in particular has a unique role in stabilizing forks stalled at hairpin structures to prevent their breakage.

### The checkpoint function of Mrc1 does not play a significant role in preventing CAG repeat fragility but is important to prevent CAG contractions

**Figure 3.**
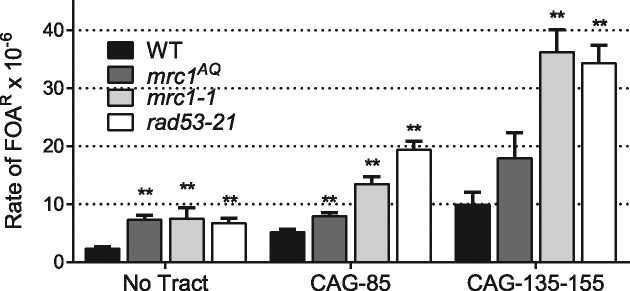
Fragility of CAG repeat tracts in checkpoint deficient mutants. *mrc1^AQ^* and *mrc1-1* strains containing a YAC with either no tract, CAG-85 medium tract, or CAG-145 (for *mrc1^AQ^*) or CAG-155 (for *mrc1-1*) long tract were assayed for their rate of FOA^R^ as in Figure 1. Data presented are an average of 3–5 experiments (Supplementary Table S1). Error bars indicate SEM. Significance compared to the WT value for the same tract length was determined using a pooled variance *t*-test, *P < 0.05; **P < 0.01.

Since Mrc1 has both a checkpoint and a stabilizer function at stalled forks, we sought to determine which function was needed for preventing fragility and instability of medium and long CAG repeats. To address this point, we used the *mrc1^AQ^* mutant in which Mrc1 is lacking its Mec1 kinase target phosphorylation sites, so that it cannot mediate checkpoint signaling but is still capable of performing its fork stabilization role of coupling the GINS complex to Polϵ (35). The *mrc1^AQ^* mutant showed only a very slight increase in fragility over WT for both medium and long CAG tracts that only reached significance for the medium tract but was still 11 times less elevated than the rate for *mrc1*Δ cells (Figure 3 and Supplementary Table S1). In fact, the fragility rate for the no tract is as high as for the medium tract, reflecting a global role rather than a role specific to CAG repeats for the checkpoint function of Mrc1. Therefore, the fork stabilization role of Mrc1, and not the checkpoint role, is responsible for preventing CAG fragility.

In our previous paper, the use of a checkpoint-deficient allele of Mrc1, *mrc1-1*, revealed a fragility phenotype for the no tract, medium, and long CAG tracts of a magnitude similar to the checkpoint-deficient *rad53-21* mutant (60). The *mrc1-1* mutant was obtained from a genetic screen to identify mutants that fail to grow in the presence of 100 mM HU and was shown to effect the checkpoint function of Mrc1p (53), however it was not clear whether the fork stabilizer function was also affected. Based on the lack of CAG-specific fragility phenotype for *mrc1^AQ^*, we suspect that the *mrc1-1* mutant has some other defect in addition to its checkpoint deficiency. Indeed, a re-test of fragility in parallel for *mrc1^AQ^, mrc1-1* and *rad53-21* mutants with both medium and long tracts confirmed that the absence of fragility phenotype is specific to the *mrc1^AQ^* mutant (Figure 3 and Supplementary Table S1). Through this analysis, we also found that the rates previously reported for *mrc1-1* and *rad53-21* (59,60) were 10-fold lower than our new data. Though we could not identify the source of the difference, we believe that the rates reported here are the correct values and that there was a calculation error in our previously reported values. Our new data confirms that fragility is increased in a *rad53-21* mutant to a level similar to or greater than that of other checkpoint mutants identified that increase CAG fragility, which include deletions of *mec1, rad9* and *rad17rad24* (9–1–1 defect) (59). Thus, though Mrc1 checkpoint function is dispensable, one of the other checkpoint pathways that signals through Rad53 is important for preventing CAG fragility.

In contrast to fragility, the checkpoint function of Mrc1 is important for preventing CAG instability, but only at the long tract. Contractions of the CAG-145 repeat were significantly increased to 43% in the *mrc1^AQ^* mutant, 2.4-fold over WT, but the 1.8-fold increase at the medium tract was not significant (Table 1, Supplementary Table S2). Expansion levels were slightly elevated, though not significantly so, and neither expansions nor contractions were as high in the *mrc1^AQ^* mutant as they were in *mrc1*Δ cells. Nevertheless, the checkpoint function of Mrc1 accounts for about half of the overall contraction and expansion frequency increase in the *mrc1Δ* mutant. In summary, our results indicate that Mrc1 prevents fragility by preventing uncoupling of the helicase and DNA polymerase at the replication fork, whereas prevention of instability (in particular, contractions) is likely mediated by both its fork stabilization and checkpoint functions.

### Mrc1 and Tof1 are needed for survival and growth of strains with expanded CAG tracts, but the Mrc1 checkpoint role is dispensable

**Figure 4.**
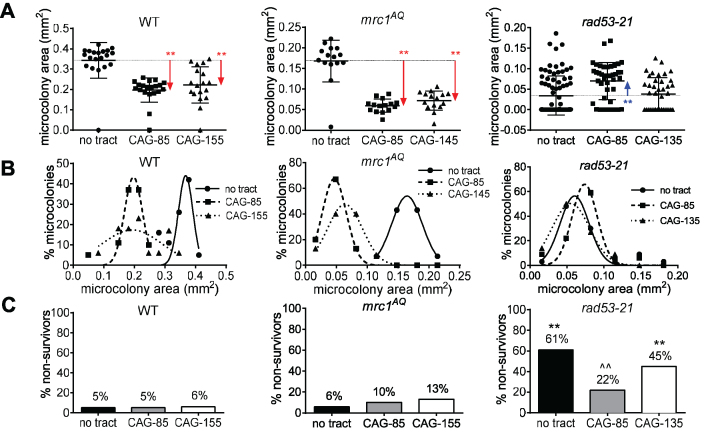
Absence of cell growth escape for the *mrc1^AQ^* mutant in the presence of expanded CAG repeats. The diameter of microcolonies after 30 h of growth on solid media was measured and converted to an area (in mm^2^). (**A**) The size distribution of all colonies is represented for WT, *mrc1^AQ^* and *rad53-21* containing either no CAG repeat, a medium tract (CAG-85) or a long tract (CAG-135, CAG-155). Note that the Y axis scales are different, as *mrc1^AQ^* and *rad53-21* strains have a smaller microcolony area on average. For each strain and each tract length, the mean and the 99% confidence intervals are represented by the horizontal bars. Comparison of the sizes of microcolonies in CAG-85 and CAG-135-155 to the no tract control of the same genotype was determined by a Fisher's LSD test, * *P* <0.05; ** *P* < 0.01; a red arrow indicates a significant decrease in the mean area compared to the no tract control; a blue arrow indicates a significant increase in the mean area compared to the no tract control. A two-way ANOVA interaction test, corrected for unequal variances, also showed a highly significant difference (df = 4, *F* = 26.1, *P* < 0.001). (**B**) Frequency size distribution of survivor cells (area ≥0.016 mm^2^ at 30 h) is depicted for the same strains as in (A). (**C**) Percentage of non-survivors (area <0.016 mm^2^ for 30 h) that arrested within the first few divisions in strains containing zero (black), 85 (gray) or 135–155 (white) CAG repeats. Significance compared to the WT value for the same tract length (*) or to the no tract value of the same strain (∧) was determined by a Fisher's exact test, * or ∧ *P* < 0.05; ** or ∧∧ *P* < 0.01. Exact *P* values and numbers of colonies analyzed are in Supplementary Table S3.

We previously demonstrated that the presence of expanded CAG-70 or CAG-155 repeats in WT yeast (BY4705 strain) causes a significant fraction of cells to undergo transient S and G_2_ phase arrests, and elicits Rad53 phosphorylation in repair-defective cells (68). The growth disadvantage of cells with expanded CAG tracts results in fewer cell divisions compared to the no tract strain. This checkpoint effect can be quantified using a microcolony assay, where single cells in log phase growth are micromanipulated onto a plate, and growth of cells into microcolonies is monitored for 30 h (68). A smaller microcolony size indicates that the checkpoint response to damage caused by the CAG tract is intact, increasing the number and the length of the cell cycle arrests (68). This is illustrated by the significantly lower colony size measured by area (Figure 4A, all microcolonies plotted) or smaller peak microcolony area (Figure 4B, only survivors plotted) of cells containing CAG-85 or CAG-155 tracts compared to the no tract control in the WT W303 strain (Figure 4A), which exhibits a profile similar to that previously found for the BY4705 strain (68). In contrast, a defective checkpoint response would relieve the arrests and allow a more normal rate of cell divisions, similar to the no tract control, which is observed in the checkpoint-deficient *rad53-21* strain background (Figure 4A and B, *P* values in Supplementary Table S3). The effect of the CAG repeat differed among genotypes (*P* < 0.001); this effect is primarily due to the loss of the CAG repeat-specific growth inhibition in the *rad53-21* mutant compared to the WT. In contrast, the *mrc1^AQ^* mutant showed a reduction in the size of the medium and long tract-containing microcolonies in a pattern similar to the corresponding WT strain (Figure 4A and 4B). This result confirms that the presence of a medium or a long CAG tract triggers a checkpoint response that does not involve the checkpoint function of Mrc1.

In addition to microcolony size, we also monitored how many of the cells failed to grow into microcolonies greater than 0.016 mm^2^ after 30 h, the non-survivors. 5–6% of W303 WT cells showed a terminal arrest after 30 hours, and the presence of medium or long CAG repeats did not affect this percentage as already observed for the BY4705 WT background (68). Cells with the *mrc1^AQ^* mutation and CAG-155 repeats had a 2-fold increase in non-survivors compared to the corresponding WT strain, consistent with the role in repair at this long repeat length that was revealed by the contraction phenotype (Table 1). This result may, at least partially, explain the decrease in average area of the *mrc1^AQ^* microcolonies compared to the WT microcolonies, and indicate an important role for the Mrc1 checkpoint function in cell growth or recovery after DNA damage, including damage at the CAG tract. The checkpoint-deficient *rad53-21* strain non-survivor percentage ranged from 22% to 61% (Figure 4C). This result reveals that cell death is a major event in the absence of the Rad53-mediated checkpoint, emphasizing the importance of the checkpoint for rescuing cells that experience DNA damage, consistent with the very small microcolony size in this background (Figure 4A). Interestingly, the presence of CAG repeats did not exacerbate the non-survivor frequency of *rad53-21* cells. On the contrary, the presence of a medium tract significantly reduced the amount of cell death (Figure 4C). We previously found that the 9–1–1 complex is important for sensing a type of damage that occurs more often at the medium CAG tract, hypothesized to be gaps due to hairpin bypass, whereas the Mec1-Ddc2-Rad53 axis is more important at long CAG tracts that efficiently stall replication (59,60). Thus, the fewer non-survivors at CAG-85 could be due to activation of a 9–1–1-dependent response that facilitates repair and prevents cell death.

Eliminating all Mrc1 function resulted in one third to one half of the microcolony population undergoing a terminal arrest. For *mrc1*Δ there were a large percentage of non-survivors: 31%, 50% and 54% for no tract, CAG-85, and CAG-155 respectively (Supplementary Figure S3A) showing the importance of Mrc1 for cell growth, a problem further exacerbated by the presence of an expanded CAG tract in the genome. The massive cell death in the *mrc1*Δ strain compared to the *mrc1^AQ^* strain demonstrates the essential role of the replicative function of Mrc1 for survival of cells with expanded CAG tracts and implies that the fragility rate and instability phenotype are likely underestimated in the *mrc1Δ* strains. The growth delay was such that even the *mrc1Δ* microcolony population that did pass the 0.016 mm^2^ area cut-off after 30 hours showed severe growth impairment with only a few that went on to form microcolonies in the 0.03–0.05 mm^2^ size range, so few that an accurate 30 h survivor size distribution could not be obtained. We extended the growth time of the *mrc1*Δ microcolony ‘non-survivors’ (e.g. that did not pass the 0.016 mm^2^ survival area cut-off after 30 h) in an attempt to obtain enough material for PCR analysis of repeat tract length. This analysis revealed a contraction event in all of the microcolonies tested. This shows that CAG instability is an event that takes place early and often at the population level in the absence of Mrc1. Similar results were observed for the *tof1*Δ strain, with 57%, 50% and 52% non-survivors for no tract, CAG-85 and CAG-155 respectively, and survivors all in the 0.03 mm^2^ size range (Supplementary Figure S3B and data not shown).

Overall, our microcolony results show that the replicative functions of Mrc1 and Tof1 as well as effective Rad53 checkpoint activation play an essential role in the successful formation of a yeast colony. Moreover, when Mrc1 is absent, the presence of an extended CAG tract becomes a significant factor that further increases the mortality rate of the cells. The absence of growth escape along with the observation of few non-survivors among the *mrc1^AQ^* microcolonies confirms that it is the Mrc1 fork stabilizer function rather than its checkpoint function that is most important for preventing DNA damage both in general and specifically at expanded CAG tracts.

## DISCUSSION

In this study, we investigated the role of Mrc1 and Tof1 at expanded CAG repeats. We revealed that both Mrc1 and Tof1 are required to stabilize the replication fork to prevent DNA breakage at the CAG repeat. In addition, their fork stabilizer function is crucial for preventing instability of the expanded CAG repeats, especially contractions. Thus, forks encountering stable DNA structures are especially reliant on stabilization by the Mrc1-Tof1-Csm3 complex to prevent fork breakage, and without this function genome instability or cell death occurs. However, discrepancies exist in the role of these two proteins at structure-forming repeats. Whereas Mrc1 exerts a protective role at all expanded CAG repeat lengths tested as well as at a control sequence that does not stall replication, Tof1 prevents chromosome fragility and replication fork progression specifically at longer CAG repeats (85 repeats and above). The specific CAG length-dependent phenotypes observed for the *tof1*Δ strain (for fragility) and the *mrc1^AQ^* checkpoint deficient strain (for contractions) point towards a difference in the type or severity of barrier generated upon formation of the hairpin at long expanded CAG repeats compared to shorter repeat tracts.

### The replicative function of Mrc1 and Tof1 are crucial for preventing fork breakage at expanded CAG repeats

Analysis of the *mrc1-1* mutant in our previous study indicated that Mrc1 prevents fragility and instability of expanded CAG repeats (60). However, it was unclear how much of the effect was due to the Mrc1 checkpoint role and how much was due to its physical interactions that couple the replisome with the MCM helicase. The new data with the full *MRC1* deletion reveal that Mrc1 has a crucial protective role at expanded CAG repeats, as the absence of Mrc1 generates a dramatic increase in CAG tract fragility. Additionally, more than half of cells containing a long CAG tract and lacking Mrc1 fail to divide more than a few times. By comparison to the fragility rate of the *mrc1^AQ^* mutant, we conclude that the checkpoint function of Mrc1 plays a minimal role in fork protection at a structure-induced stall compared to its function in coupling Polϵ to the GINS complex and MCM helicase. A fragility phenotype was also demonstrated for expanded triplex-forming GAA repeats in *mrc1*Δ strains (33) and fork stalling was also increased in *mrc1*Δ or *tof1*Δ strains at hairpin-forming CGG repeats and inverted repeats (9,10). Thus, Mrc1 exerts an important protective role at forks stalled by various DNA structure impediments. By maintaining a tight replisome, Mrc1 could block access of the DNA to Exo1, thus minimizing ssDNA and resultant breaks (69). An additional factor is that the extra ssDNA generated in *mrc1*Δ cells could affect the size or stability of the hairpin, thus reinforcing the fork stall and increasing fragility. The increase in replication fork slowing % at the CAG-130 tract is supportive of this idea, though stalling at the CAG-70 tract did not become evident. At CAG/CTG repeats, Msh2 stabilization of hairpins increases fork stalling (25). Thus, by coupling the helicase to the replicase, Mrc1 could reduce the likelihood of hairpin formation or the access of Msh2 to stabilize those hairpins, indirectly reducing fork stalling. Alternatively, a recent study shows that Mrc1 can directly stimulate Polϵ synthesis (70), and Pol2 levels at the replication fork are substantially decreased in *mrc1*Δ cells (35), which could lead to a failure to restart replication after a fork stall.

Surprisingly, the absence of Tof1 generates a dramatic fragility phenotype only for the expanded CAG repeats of long length (110–155 repeat units), which implies that different mechanisms, reflecting different substrates, are at play to stabilize a fork encountering a medium versus a long CAG tract. Several lines of evidence point to a change in DNA damage upon lengthening of structure-forming repeat tracts that could influence the fragility outcome of the fork. First, forks encountering ∼55 CTG repeats generally do not form a clearly visible stall on a 2D gel, but there is substantial formation of joint molecules migrating in a cone shape off the Y arc that likely include resected reversed forks (24,26). When the CAG/CTG tract size increased to 80 on a plasmid or 98–120 repeats on a yeast chromosome, a shift occurred with fewer joint molecules and a more discrete pausing signal visible (23,25,26). These 2D data suggest that the stall is more difficult to recover from as CAG tracts reach around 80–100 repeat units. The 2D gel data in Figure 2 reinforce this conclusion, as a visible stall was only detected at 130, not 70 repeats, and the absence of Tof1 further increased replication fork stalling at CAG-130. Second, CAG tracts of 130 repeats relocate more frequently to the nuclear pore than CAG tracts of 70 repeats (71). The movement to the nuclear periphery occurs for collapsed forks or hard-to-repair double-strand breaks (72). Altogether, these data suggest that larger or more frequently formed hairpins at long expanded CAG repeats impose a stronger barrier to replication, leading to a fork-stalling event that requires Tof1. For example, through its physical interaction with the Polα, Tof1 could provide coupling between the lagging strand replisome and MCM (35,40,73). This idea is supported by data that a primase mutant (*pri2-1*) also exhibits a greater fragility phenotype for long expanded CAG repeats (30,74). The stable stall could be generated either due to a CAG hairpin on the lagging strand template or a CTG hairpin formed on the leading strand template (or both) reaching a threshold size that can no longer be bypassed.

### The role of Mrc1 and Tof1 in maintaining the stability of expanded CAG repeats

Our results highlight the importance of the Mrc1 fork protection function in preventing repeat expansions of medium to long CAG tracts that stall replication (85–155 repeats), as we observed a highly significant increase in expansions in *mrc1*Δ cells even though contractions were extremely frequent. Since the checkpoint-deficient *mrc1^AQ^* allele had a lesser effect, we conclude that the Polϵ replicase–helicase coupling is vital for preventing expansion of long repetitive tracts. In the absence of Mrc1, the DNA could more easily transition to a hairpin on the nascent strand or to a reversed fork to allow hairpin formation on the nascent reversed strand, leading to an expansion event if the hairpin is incorporated, as initially proposed by (75). Alternatively, the addition of extra CAG tracts could occur during a template switch event after bypass of a hairpin, or during the DSB repair process after fork collapse (16,76,77). Mrc1 coupling could also facilitate the unwinding of the hairpin by Srs2 or Sgs1 helicases (11,24,26,78). In human cell lines, siRNA knockdown of Claspin, Timeless or Tipin increases the occurrence of expansion at CAG or CTG tracts of 100 repeats (79). Thus, the importance of fork coupling to prevent repeat expansions is a conserved feature between yeast and human cells. The additional requirement of the Timeless/Tipin complex to prevent expansions in human cells suggests that Timeless may have acquired a greater function through evolution. We note that, using a sensitive genetic assay, a role for Tof1 in preventing the expansion of GAA repeats, which form a triplex structure, or ATTCT repeats, which do not form a structure, was reported (51,80). Therefore Tof1 may aid in preventing expansions at some repeats, but at CAG tracts Mrc1 has the greater role. In contrast to expansions, Mrc1 and Tof1 are equally important for preventing CAG-85 and CAG-155 contractions. In human cells, both Claspin and Timeless/Tipin are also required to prevent the contraction of expanded CAG or CTG repeats (79). Since *mrc1*Δ and *tof1*Δ strains had a significant fragility phenotype for these lengths, contractions could occur due to misalignments during repair of the broken forks. A break within such a long repetitive tract favors a single strand annealing (SSA) repair pathway, which will produce a contraction event (76,81).

A previous study on CAG/CTG repeats at a sub-threshold size of 13 repeats attributed a role for Tof1 and the checkpoint function of Mrc1 in inhibiting expansions (61), which we did not detect. This could reflect a difference in assay sensitivity, as the genetic assay used for detecting expansions from (CTG)_13_ can detect very low frequency events. Although not significant, the *mrc1^AQ^* and *rad53-21* mutants induced a 2.5- to 4-fold increase in CAG expansions over the WT at the CAG-85 repeat. Alternatively, the different CAG orientations used in the two assays could play a role (CTG (61) versus CAG (here) on the lagging strand template). Analysis of a CAG-120 repeat by 2D gel showed that stalling is more pronounced when the stronger CTG hairpin is on the leading strand template (as in our case), compared to when CTG is on the lagging strand template (25). Thus the nature of fork progression could be different in the two orientations and invoke a different response. For example, a CTG lagging strand hairpin could be bypassed, leading to a ssDNA gap that induces an Mrc1-dependent checkpoint response, while a CTG hairpin on the leading strand template could more effectively stall the replisome, requiring fork coupling and restart mechanisms. Consistent with this idea, the replication checkpoint played a significant role in preventing contractions (*rad53-21* at both tract lengths and *mrc1^AQ^* at (CAG)_145_), even though effects on fragility were much more modest. A possible mechanism of generating contractions is slippage at single-strand DNA, which accumulates at stalled forks in checkpoint-deficient mutants due to the resection action of Exo1 (82–86).

## CONCLUSIONS

In conclusion, the fragility and instability phenotype differences obtained for *tof1*Δ and *mrc1*Δ in our assays suggest that the Tof1 replicative function exerts its protective role when fork stalling is severe enough to require extra stabilization, such as at long expanded CAG repeats. In contrast, Mrc1 exerts its fork stability at all forks via its helicase-replicase coupling function but is especially crucial at DNA structure-induced fork barriers. Mrc1 and Tof1 are both critical for cell survival, as more than half of cells lacking one of these proteins examined were not able to complete more than a few cell divisions. Comparison of the *mrc1*Δ, *mrc1^AQ^* and *rad53-21* mutants revealed that the DNA damage checkpoint regulated by Rad53 (hChk2) is important for promoting cell division and preventing chromosome fragility. However, the replication checkpoint mediated by Mrc1 has a minimal role in preventing fork breakage, though it is important for preventing CAG instability, especially contractions. Altogether, our results reveal a complex interplay of events at stalled replication forks, with each component of the Mrc1–Tof1–Csm3 (Claspin–Timeless–Tipin) complex playing a unique role in protecting against repeat instability and fork collapse. The conserved role of Tof1 and Mrc1 through evolution underlines the importance of maintaining replication fork architecture to avoid breaks at DNA structures and resulting genome instability.

## Supplementary Material

Supplementary Data

## References

[1] Hyrien O. Mechanisms and consequences of replication fork arrest. Biochimie. 2000; 82:5–17. 10717381 10.1016/s0300-9084(00)00344-8

[2] Mirkin E. V., Mirkin S.M. Replication fork stalling at natural impediments. Microbiol. Mol. Biol. Rev. 2007; 71:13–35. 17347517 10.1128/MMBR.00030-06 PMC1847372

[3] Aguilera A., García-Muse T. Causes of genome instability. Annu. Rev. Genet. 2013; 47:1–32. 23909437 10.1146/annurev-genet-111212-133232

[4] Bartkova J., Rezaei N., Liontos M., Karakaidos P., Kletsas D., Issaeva N., Vassiliou L.-V.F., Kolettas E., Niforou K., Zoumpourlis V.C. Oncogene-induced senescence is part of the tumorigenesis barrier imposed by DNA damage checkpoints. Nature. 2006; 444:633–637. 17136093 10.1038/nature05268

[5] Di Micco R., Fumagalli M., Cicalese A., Piccinin S., Gasparini P., Luise C., Schurra C., Garre’ M., Giovanni Nuciforo P., Bensimon A. Oncogene-induced senescence is a DNA damage response triggered by DNA hyper-replication. Nature. 2006; 444:638–642. 17136094 10.1038/nature05327

[6] Halazonetis T.D., Gorgoulis V.G., Bartek J. An Oncogene-Induced DNA damage model for cancer development. Science. 2008; 319:1352–1355. 18323444 10.1126/science.1140735

[7] Sarni Dan, Kerem B Oncogene-Induced replication stress drives genome instability and tumorigenesis. Int. J. Mol. Sci. 2017; 18:1339.

[8] Krasilnikova M.M., Mirkin S.M. Replication stalling at Friedreich's Ataxia (GAA)n repeats in vivo. Mol. Cell. Biol. 2004; 24:2286–2295. 14993268 10.1128/MCB.24.6.2286-2295.2004 PMC355872

[9] Voineagu I., Narayanan V., Lobachev K.S., Mirkin S.M. Replication stalling at unstable inverted repeats: Interplay between DNA hairpins and fork stabilizing proteins. Proc. Natl. Acad. Sci. U.S.A. 2008; 105:9936–9941. 18632578 10.1073/pnas.0804510105 PMC2481305

[10] Voineagu I., Surka C.F., Shishkin A.A., Krasilnikova M.M., Mirkin S.M. Replisome stalling and stabilization at CGG repeats, which are responsible for chromosomal fragility. Nat. Struct. Mol. Biol. 2009; 16:226–228. 19136957 10.1038/nsmb.1527 PMC2837601

[11] Anand R.P., Shah K.A., Niu H., Sung P., Mirkin S.M., Freudenreich C.H. Overcoming natural replication barriers: differential helicase requirements. Nucleic Acids Res. 2012; 40:1091–1105. 21984413 10.1093/nar/gkr836 PMC3273818

[12] Lu S., Wang G., Bacolla A., Zhao J., Spitser S., Vasquez K.M. Short inverted repeats are hotspots for genetic instability: Relevance to cancer genomes. Cell Rep. 2015; 10:1674–1680. 10.1016/j.celrep.2015.02.039 PMC6013304 25772355

[13] Lai P.J., Lim C.T., Le H.P., Katayama T., Leach D.R.F., Furukohri A., Maki H. Long inverted repeat transiently stalls DNA replication by forming hairpin structures on both leading and lagging strands. Genes Cells. 2016; 21:136–145. 26738888 10.1111/gtc.12326

[14] Chandok G.S., Patel M.P., Mirkin S.M., Krasilnikova M.M. Effects of Friedreich's ataxia GAA repeats on DNA replication in mammalian cells. Nucleic Acids Res. 2012; 40:3964–3974. 22262734 10.1093/nar/gks021 PMC3351192

[15] Follonier C., Oehler J., Herrador R., Lopes M. Friedreich's ataxia–associated GAA repeats induce replication-fork reversal and unusual molecular junctions. Nat. Struct. Mol. Biol. 2013; 20:486–494. 23454978 10.1038/nsmb.2520

[16] Usdin K., Kumari D. Repeat-mediated epigenetic dysregulation of the FMR1 gene in the fragile X-related disorders Frontiers Media SA. Front Genet. 2015; 6:192. 26089834 10.3389/fgene.2015.00192 PMC4452891

[17] Paulson H. Repeat expansion diseases. Handb. Clin. Neurol. 2018; 147:105–123. 29325606 10.1016/B978-0-444-63233-3.00009-9 PMC6485936

[18] Marquis Gacy A., Goellner G., Juranić N., Macura S., McMurray C.T. Trinucleotide repeats that expand in human disease form hairpin structures in vitro. Cell. 1995; 81:533–540. 7758107 10.1016/0092-8674(95)90074-8

[19] Mitas M. Trinucleotide repeats associated with human disease. Nucleic Acids Res. 1997; 25:2245–2254. 9171073 10.1093/nar/25.12.2245 PMC146772

[20] Gacy A.M., McMurray C.T. Influence of hairpins on template reannealing at trinucleotide repeat duplexes: A model for slipped DNA. Biochemistry. 1998; 37:9426–9434. 9649325 10.1021/bi980157s

[21] Santhana Mariappan S.V., Silks L.A., Chen X., Springer P.A., Wu R., Moyzis R.K., Bradbury E.M., Garcia A.E., Gupta G. Solution structures of the huntington's disease dna triplets, (cag)n. J. Biomol. Struct. Dyn. 1998; 15:723–744. 9514249 10.1080/07391102.1998.10508988

[22] Liu G., Chen X., Bissler J.J., Sinden R.R., Leffak M. Replication-dependent instability at (CTG) x (CAG) repeat hairpins in human cells. Nat. Chem. Biol. 2010; 6:652–659. 20676085 10.1038/nchembio.416 PMC2924473

[23] Pelletier R., Krasilnikova M.M., Samadashwily G.M., Lahue R., Mirkin S.M. Replication and expansion of trinucleotide repeats in yeast. Mol. Cell. Biol. 2003; 23:1349–1357. 12556494 10.1128/MCB.23.4.1349-1357.2003 PMC141142

[24] Kerrest A., Anand R.P., Sundararajan R., Bermejo R., Liberi G., Dujon B., Freudenreich C.H., Richard G.-F. SRS2 and SGS1 prevent chromosomal breaks and stabilize triplet repeats by restraining recombination. Nat. Struct. Mol. Biol. 2009; 16:159–167. 19136956 10.1038/nsmb.1544 PMC4454460

[25] Viterbo D., Michoud G., Mosbach V., Dujon B., Richard G.-F. Replication stalling and heteroduplex formation within CAG/CTG trinucleotide repeats by mismatch repair. DNA Repair (Amst). 2016; 42:94–106. 27045900 10.1016/j.dnarep.2016.03.002

[26] Nguyen J.H.G., Viterbo D., Anand R.P., Verra L., Sloan L., Richard G.-F., Freudenreich C.H. Differential requirement of Srs2 helicase and Rad51 displacement activities in replication of hairpin-forming CAG/CTG repeats. Nucleic Acids Res. 2017; 45:4519–4531. 28175398 10.1093/nar/gkx088 PMC5416882

[27] Liu G., Chen X., Gao Y., Lewis T., Barthelemy J., Leffak M. Altered replication in human cells promotes DMPK (CTG)n {middle dot} (CAG)n repeat instability. Mol. Cell. Biol. 2012; 32:1618–1632. 22354993 10.1128/MCB.06727-11 PMC3347245

[28] Fouché N., Ozgür S., Roy D., Griffith J.D. Replication fork regression in repetitive DNAs. Nucleic Acids Res. 2006; 34:6044–6050. 17071963 10.1093/nar/gkl757 PMC1635326

[29] Freudenreich C.H., Kantrow S.M., Zakian V.A. Expansion and length-dependent fragility of CTG repeats in yeast. Science. 1998; 279:853–856. 9452383 10.1126/science.279.5352.853

[30] Callahan J.L., Andrews K.J., Zakian V.A., Freudenreich C.H. Mutations in yeast replication proteins that increase CAG / CTG expansions also increase repeat fragility. Mol. Cell. Biol. 2003; 23:7849–7860. 14560028 10.1128/MCB.23.21.7849-7860.2003 PMC207578

[31] Balakumaran B.S., Freudenreich C.H., Zakian V.A. CGG/CCG repeats exhibit orientation-dependent instability and orientation-independent fragility in Saccharomyces cerevisiae. Hum. Mol. Genet. 2000; 9:93–100. 10587583 10.1093/hmg/9.1.93

[32] Kim H.-M.M., Narayanan V., Mieczkowski P.A., Petes T.D., Krasilnikova M.M., Mirkin S.M., Lobachev K.S. Chromosome fragility at GAA tracts in yeast depends on repeat orientation and requires mismatch repair. EMBO J. 2008; 27:2896–2906. 18833189 10.1038/emboj.2008.205 PMC2580784

[33] Zhang Y., Shishkin A.A., Nishida Y., Marcinkowski-Desmond D., Saini N., Volkov K.V., Mirkin S.M., Lobachev K.S. Genome-wide screen identifies pathways that govern GAA/TTC repeat fragility and expansions in dividing and nondividing yeast cells. Mol. Cell. 2012; 48:254–265. 22959270 10.1016/j.molcel.2012.08.002 PMC3635072

[34] Nedelcheva M.N., Roguev A., Dolapchiev L.B., Shevchenko A.A., Taskov H.B., Shevchenko A.A., Stewart A.F., Stoynov S.S. Uncoupling of unwinding from DNA synthesis implies regulation of MCM helicase by Tof1/Mrc1/Csm3 checkpoint complex. J. Mol. Biol. 2005; 347:509–521. 15755447 10.1016/j.jmb.2005.01.041

[35] Lou H., Komata M., Katou Y., Guan Z., Reis C.C., Budd M., Shirahige K., Campbell J.L. Mrc1 and DNA polymerase epsilon function together in linking DNA replication and the S phase checkpoint. Mol. Cell. 2008; 32:106–117. 18851837 10.1016/j.molcel.2008.08.020 PMC2699584

[36] Komata M., Bando M., Araki H., Shirahige K. The direct binding of Mrc1, a checkpoint mediator, to Mcm6, a replication helicase, is essential for the replication checkpoint against Methyl Methanesulfonate-Induced stress. Mol. Cell. Biol. 2009; 29:5008–5019. 19620285 10.1128/MCB.01934-08 PMC2738294

[37] Serçin Ö., Kemp M.G. Characterization of functional domains in human Claspin. Cell Cycle. 2011; 10:1599–1606. 21478680 10.4161/cc.10.10.15562 PMC3127160

[38] Cho W.-H., Kang Y.-H., An Y.-Y., Tappin I., Hurwitz J., Lee J.-K. Human Tim-Tipin complex affects the biochemical properties of the replicative DNA helicase and DNA polymerases. Proc. Natl. Acad. Sci. U.S.A. 2013; 110:2523–2527. 23359676 10.1073/pnas.1222494110 PMC3574903

[39] Bastia D., Srivastava P., Zaman S., Choudhury M., Mohanty B.K., Bacal J., Langston L.D., Pasero P., O’Donnell M.E. Phosphorylation of CMG helicase and Tof1 is required for programmed fork arrest. Proc. Natl. Acad. Sci. U.S.A. 2016; 113:E3639–E3648. 27298353 10.1073/pnas.1607552113 PMC4932992

[40] Errico A., Cosentino C., Rivera T., Losada A., Schwob E., Hunt T., Costanzo V. Tipin/Tim1/And1 protein complex promotes Polα chromatin binding and sister chromatid cohesion. EMBO J. 2009; 28:3681–3692. 19893489 10.1038/emboj.2009.304 PMC2775894

[41] Kemp M.G., Akan Z., Yilmaz S., Grillo M., Smith-Roe S.L., Kang T.H., Cordeiro-Stone M., Kaufmann W.K., Abraham R.T., Sancar A. Tipin-replication protein A interaction mediates Chk1 phosphorylation by ATR in response to genotoxic stress. J. Biol. Chem. 2010; 285:16562–16571. 20233725 10.1074/jbc.M110.110304 PMC2878033

[42] Gotter A.L., Suppa C., Emanuel B.S. Mammalian TIMELESS and Tipin are evolutionarily conserved replication fork-associated factors. J. Mol. Biol. 2007; 366:36–52. 17141802 10.1016/j.jmb.2006.10.097 PMC4151250

[43] Katou Y., Kanoh Y., Bando M., Noguchi H., Tanaka H., Ashikari T., Sugimoto K., Shirahige K. S-phase checkpoint proteins Tof1 and Mrc1 form a stable replication-pausing complex. Nature. 2003; 424:1078–1083. 12944972 10.1038/nature01900

[44] Tourrière H., Versini G., Cordón-Preciado V., Alabert C., Pasero P. Mrc1 and Tof1 promote replication fork progression and recovery independently of Rad53. Mol. Cell. 2005; 19:699–706. 16137625 10.1016/j.molcel.2005.07.028

[45] Hodgson B., Calzada A., Labib K. Mrc1 and Tof1 Regulate DNA replication forks in different ways during normal S phase. Mol. Biol. Cell. 2007; 18:3894–3902. 17652453 10.1091/mbc.E07-05-0500 PMC1995724

[46] Yeeles J.T.P.P., Janska A., Early A., Diffley J.F.X.X. How the eukaryotic replisome achieves rapid and efficient DNA replication. Mol. Cell. 2017; 65:105–116. 27989442 10.1016/j.molcel.2016.11.017 PMC5222725

[47] Bando M., Katou Y., Komata M., Tanaka H., Itoh T., Sutani T., Shirahige K. Csm3, Tof1, and Mrc1 form a heterotrimeric mediator complex that associates with DNA replication forks. J. Biol. Chem. 2009; 284:34355–34365. 19819872 10.1074/jbc.M109.065730 PMC2797203

[48] Uzunova S.D., Zarkov A.S., Ivanova A.M., Stoynov S.S., Nedelcheva-Veleva M.N. The subunits of the S-phase checkpoint complex Mrc1/Tof1/Csm3: Dynamics and interdependence. Cell Div. 2014; 9:1–15. 25379053 10.1186/1747-1028-9-4 PMC4221646

[49] Bjergbaek L., Cobb J.A., Tsai-Pflugfelder M., Gasser S.M. Mechanistically distinct roles for Sgs1p in checkpoint activation and replication fork maintenance. EMBO J. 2005; 24:405–417. 15616582 10.1038/sj.emboj.7600511 PMC545806

[50] Pardo B., Crabbé L., Pasero P. Signaling pathways of replication stress in yeast. FEMS Yeast Res. 2017; 17:doi:10.1093/femsyr/fow101. 10.1093/femsyr/fow101 27915243

[51] Cherng N., Shishkin A.A., Schlager L.I., Tuck R.H., Sloan L., Matera R., Sarkar P.S., Ashizawa T., Freudenreich C.H., Mirkin S.M. Expansions, contractions, and fragility of the spinocerebellar ataxia type 10 pentanucleotide repeat in yeast. Proc. Natl. Acad. Sci. U.S.A. 2011; 108:2843–2848. 21282659 10.1073/pnas.1009409108 PMC3041125

[52] Mohanty B.K., Bairwa N.K., Bastia D. The Tof1p-Csm3p protein complex counteracts the Rrm3p helicase to control replication termination of Saccharomyces cerevisiae. Proc. Natl. Acad. Sci. U.S.A. 2006; 103:897–902. 16418273 10.1073/pnas.0506540103 PMC1347974

[53] Alcasabas A.A., Osborn A.J., Bachant J., Hu F., Werler P.J.H., Bousset K., Furuya K., Diffley J.F.X., Carr A.M., Elledge S.J. Mrc1 transduces signals of DNA replication stress to activate Rad53. Nat. Cell Biol. 2001; 3:958–965. 11715016 10.1038/ncb1101-958

[54] Osborn A.J., Elledge S.J. response to DNA replication stress activates Rad53 Mrc1 is a replication fork component whose phosphorylation in response to DNA replication stress activates Rad53. Genes Dev. 2003; 17:1755–1767. 12865299 10.1101/gad.1098303 PMC196183

[55] Tanaka K., Russell P. Mrc1 channels the DNA replication arrest signal to checkpoint kinase Cds1. Nat. Cell Biol. 2001; 3:966–972. 11715017 10.1038/ncb1101-966

[56] Foss E.J. Tof1p regulates DNA damage responses during S phase in Saccharomyces cerevisiae. Genetics. 2001; 157:567–577. 11156979 10.1093/genetics/157.2.567 PMC1461533

[57] Noguchi E., Noguchi C., Du L.-L., Russell P. Swi1 prevents replication fork collapse and controls checkpoint kinase Cds1. Mol. Cell. Biol. 2003; 23:7861–7874. 14560029 10.1128/MCB.23.21.7861-7874.2003 PMC207622

[58] Noguchi E., Noguchi C., McDonald W.H., Yates J.R., Russell P. Swi1 and Swi3 are components of a replication fork protection complex in fission yeast. Mol. Cell. Biol. 2004; 24:8342–8355. 15367656 10.1128/MCB.24.19.8342-8355.2004 PMC516732

[59] Lahiri M., Gustafson T.L., Majors E.R., Freudenreich C.H. Expanded CAG repeats activate the DNA damage checkpoint pathway. Mol. Cell. 2004; 15:287–293. 15260979 10.1016/j.molcel.2004.06.034

[60] Freudenreich C.H., Lahiri M. Structure-forming CAG/CTG repeat sequences are sensitive to breakage in the absence of Mrc1 checkpoint function and S-phase checkpoint signaling: implications for trinucleotide repeat expansion diseases. Cell Cycle. 2004; 3:1370–1374. 15483399 10.4161/cc.3.11.1246

[61] Razidlo D.F., Lahue R.S. Mrc1, Tof1 and Csm3 inhibit CAG·CTG repeat instability by at least two mechanisms. DNA Repair (Amst). 2008; 7:633–640. 18321795 10.1016/j.dnarep.2008.01.009 PMC2396238

[62] Brachmann C.B., Davies A., Cost G.J., Caputo E., Li J., Hieter P., Boeke J.D. Designer deletion strains derived from Saccharomyces cerevisiae S288C: a useful set of strains and plasmids for PCR-mediated gene disruption and other applications. Yeast. 1998; 14:115–132. 9483801 10.1002/(SICI)1097-0061(19980130)14:2<115::AID-YEA204>3.0.CO;2-2

[63] Longtine M.S., McKenzie A., Demarini D.J., Shah N.G., Wach A., Brachat A., Philippsen P., Pringle J.R. Additional modules for versatile and economical PCR-based gene deletion and modification in Saccharomyces cerevisiae. Yeast. 1998; 14:953–961. 9717241 10.1002/(SICI)1097-0061(199807)14:10<953::AID-YEA293>3.0.CO;2-U

[64] Dutcher S.K. Internuclear transfer of genetic information in kar1-1/KAR1 heterokaryons in Saccharomyces cerevisiae. Mol. Cell. Biol. 1981; 1:245–253. 6765600 10.1128/mcb.1.3.245-253.1981 PMC369668

[65] Polleys E.J., Freudenreich C.H. Methods to Study Repeat Fragility and Instability in Saccharomyces cerevisiae. Methods Mol. Biol. 2018; 1672:403–419. 29043639 10.1007/978-1-4939-7306-4_28

[66] Zheng Q. Statistical and algorithmic methods for fluctuation analysis with SALVADOR as an implementation. Math. Biosci. 2002; 176:237–252. 11916511 10.1016/s0025-5564(02)00087-1

[67] Gietz R.D., Woods R.A. Transformation of yeast by lithium acetate/single-stranded carrier DNA/polyethylene glycol method. Methods Enzymol. 2002; 350:87–96. 12073338 10.1016/s0076-6879(02)50957-5

[68] Sundararajan R., Freudenreich C.H. Expanded CAG/CTG repeat DNA induces a checkpoint response that impacts cell proliferation in Saccharomyces cerevisiae. PLoS Genet. 2011; 7:e1001339. 21437275 10.1371/journal.pgen.1001339 PMC3060079

[69] Sasaki M., Kobayashi T. Ctf4 prevents genome rearrangements by suppressing DNA Double-Strand break formation and its end resection at arrested replication forks. Mol. Cell. 2017; 66:533–545. 28525744 10.1016/j.molcel.2017.04.020

[70] Zhang Z.-X.X., Zhang J., Cao Q., Campbell J.L., Lou H. The DNA Pol ε stimulatory activity of Mrc1 is modulated by phosphorylation. Cell Cycle. 2018; 17:64–72. 29157061 10.1080/15384101.2017.1403680 PMC5815433

[71] Su X.A., Dion V., Gasser S.M., Freudenreich C.H. Regulation of recombination at yeast nuclear pores controls repair and triplet repeat stability. Genes Dev. 2015; 29:1006–1017. 25940904 10.1101/gad.256404.114 PMC4441049

[72] Freudenreich C.H., Su X.A. Relocalization of DNA lesions to the nuclear pore complex. FEMS Yeast Res. 2016; 16:1–9. 10.1093/femsyr/fow095 PMC5113167 27799300

[73] Branzei D., Foiani M. The DNA damage response during DNA replication. Curr. Opin. Cell Biol. 2005; 17:568–575. 16226452 10.1016/j.ceb.2005.09.003

[74] Sundararajan R., Gellon L., Zunder R.M., Freudenreich C.H. Double-Strand break repair pathways protect against CAG/CTG repeat expansions, contractions and Repeat-Mediated chromosomal fragility in saccharomyces cerevisiae. Genetics. 2010; 184:65–77. 19901069 10.1534/genetics.109.111039 PMC2815931

[75] Mirkin S.M. DNA structures, repeat expansions and human hereditary disorders. Curr. Opin. Struct. Biol. 2006; 16:351–358. 16713248 10.1016/j.sbi.2006.05.004

[76] Polleys E.J., House N.C.M., Freudenreich C.H. Role of recombination and replication fork restart in repeat instability. DNA Repair (Amst). 2017; 56:156–165. 28641941 10.1016/j.dnarep.2017.06.018 PMC5541998

[77] Kim J.C., Mirkin S.M. The balancing act of DNA repeat expansions. Curr. Opin. Genet. Dev. 2013; 23:280–288. 23725800 10.1016/j.gde.2013.04.009 PMC3703482

[78] Bhattacharyya S., Lahue R.S. Srs2 helicase of Saccharomyces cerevisiae selectively unwinds triplet repeat DNA. J. Biol. Chem. 2005; 280:33311–33317. 16085654 10.1074/jbc.M503325200

[79] Liu G., Chen X., Gao Y., Lewis T., Barthelemy J., Leffak M. Altered replication in human cells promotes DMPK (CTG)(n) · (CAG)(n) repeat instability. Mol. Cell. Biol. 2012; 32:1618–1632. 22354993 10.1128/MCB.06727-11 PMC3347245

[80] Shishkin A.A., Voineagu I., Matera R., Cherng N., Chernet B.T., Krasilnikova M.M., Narayanan V., Lobachev K.S., Mirkin S.M. Large-Scale expansions of Friedreich's ataxia GAA repeats in Yeast. Mol. Cell. 2009; 35:82–92. 19595718 10.1016/j.molcel.2009.06.017 PMC2722067

[81] Mosbach V., Poggi L., Viterbo D., Charpentier M., Richard G.-F. TALEN-Induced Double-Strand break repair of CTG trinucleotide repeats. Cell Rep. 2018; 22:2146–2159. 29466740 10.1016/j.celrep.2018.01.083

[82] Sogo J.M., Lopes M., Foiani M. Fork reversal and ssDNA accumulation at stalled replication forks owing to checkpoint defects. Science. 2002; 297:599–602. 12142537 10.1126/science.1074023

[83] Cotta-Ramusino C., Fachinetti D., Lucca C., Doksani Y., Lopes M., Sogo J., Foiani M. Exo1 processes stalled replication forks and counteracts fork reversal in checkpoint-defective cells. Mol. Cell. 2005; 17:153–159. 15629726 10.1016/j.molcel.2004.11.032

[84] Sabatinos S.A., Forsburg S.L. Managing single-stranded DNA during replication stress in fission yeast. Biomolecules. 2015; 5:2123–2139. 26393661 10.3390/biom5032123 PMC4598791

[85] Colosio A., Frattini C., Pellicanò G., Villa-Hernández S., Bermejo R. Nucleolytic processing of aberrant replication intermediates by an Exo1-Dna2-Sae2 axis counteracts fork collapse-driven chromosome instability. Nucleic Acids Res. 2016; 44:10676–10690. 27672038 10.1093/nar/gkw858 PMC5159547

[86] Iyer D.R., Rhind N. Replication fork slowing and stalling are distinct, checkpoint-independent consequences of replicating damaged DNA. PLoS Genet. 2017; 13:e1006958. 28806726 10.1371/journal.pgen.1006958 PMC5570505

