# Supplementary material for: Mrc1 and Tof1 prevent fragility and instability at long CAG repeats by their fork stabilizing function
Source: Nucleic Acids Res. 2018 Nov 23;47(2):794–805. doi: 10.1093/nar/gky1195 (PMC6344861; doi:10.1093/nar/gky1195)
Supplement: Supplementary Data [file gky1195_supplemental_files.docx]

**Gellon et al. Supplementary Data**

**Supplemental Figures**


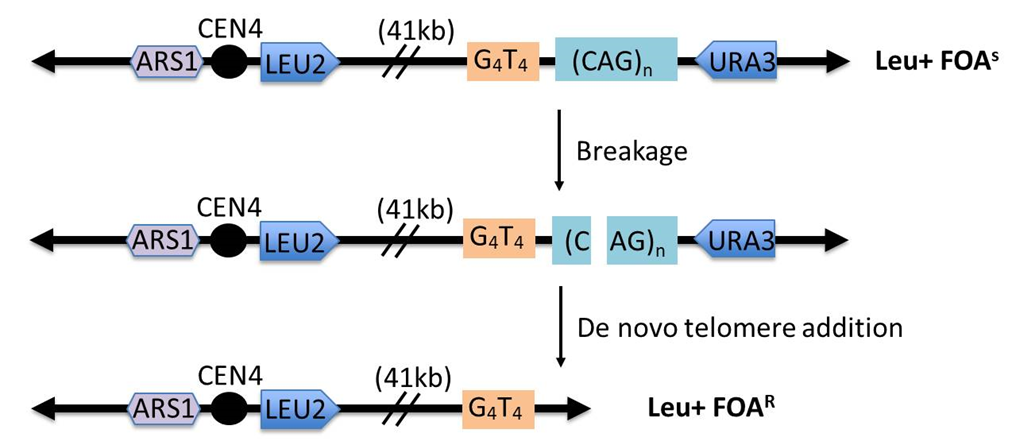


**Figure S1. YAC end loss assay used to measure CAG Fragility.**

Rates of FOA^R^ are determined as described in the Methods. When the YAC undergoes breakage at or near the CAG repeats, it can result in loss of the *URA3* gene and resistance to 5-fluoroorotic acid (FOA^R^). Resection to the G_4_T_4_ telomere seed sequence followed by telomere addition results in stabilization of the YAC; cells that are Leu+ and FOA^R^ are selected by plating on YC-Leu+FOA. Telomeres are represented by an arrow and the centromere by a black circle. Typically 95-100% of FOA^R^ colonies are due to end loss for YACs containing CAG-70 to CAG-155, indicating chromosomal breakage of DNA sequences containing expanded CAG repeats (30, 75) and unpublished data).


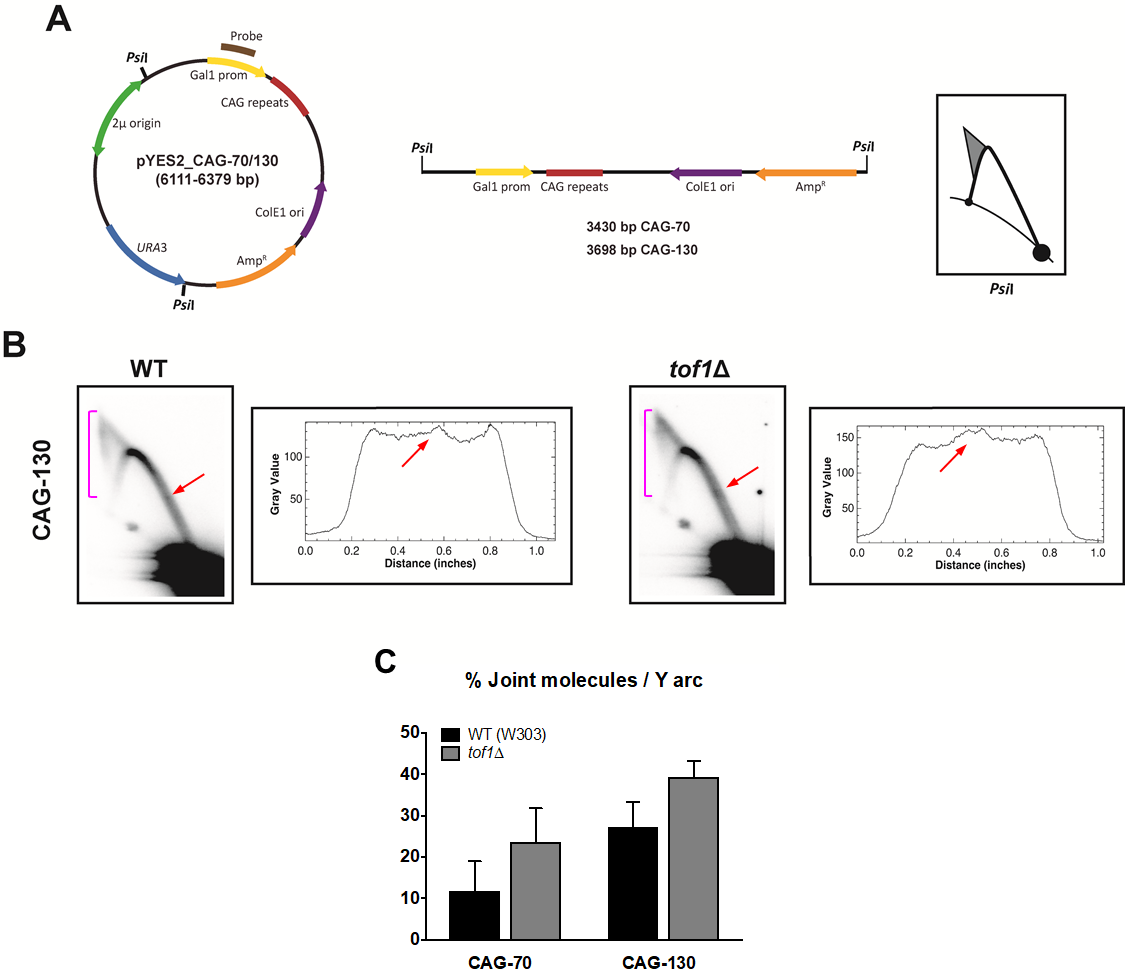


**Figure S2. Analysis of replication through CAG-130 repeats by two-dimensional (2D) agarose gel electrophoresis in WT and *tof1*∆ cells.**

(A) Schematic of the pYES2_CAG-130 is shown with its mass and genetic map. The relative positions of its most relevant features are indicated inside: the 2 micron origin, the ColE1 unidirectional origin (ColE1 Ori), the ampicillin-resistance gene (Amp^R^), *URA3*, the promoter of galactose (Gal1 prom) and 70 or 130 CAG repeats. Outside, the relative positions of sites recognized by the restriction endonuclease *Psi*I are indicated. To the right is the corresponding linear map of the *Psi*I restriction fragment with the sizes and the diagrammatic interpretation if replication initiates bi-directionally at the 2 micron origin. (B) Representative 2D gels of replication through CAG-130 in WT and *tof1*Δ strains. DNA was isolated, digested with *Psi*I and analyzed by 2D gels. To the right of both 2D gels are shown the densitometric profiles corresponding to the Y-arc region where the CAG-130 repeats are cloned. Red arrow points to the location of the stall due to the CAG repeats. Pink square brackets indicate joint molecules. (C) Quantification of the signal of the joint molecules in pYES2_CAG-70 and CAG-130 in WT and *tof1*Δ strains. Joint molecule signals were normalized to the Y arc. Two different experiments were performed in each case (average of joint molecules for each condition: CAG-70 WT 14.7 %, CAG-70 *tof1*Δ 23.5 %, CAG-130 WT 27.2 % and CAG-130 *tof1*Δ 39.3 %). Error bars indicate standard error of the mean.


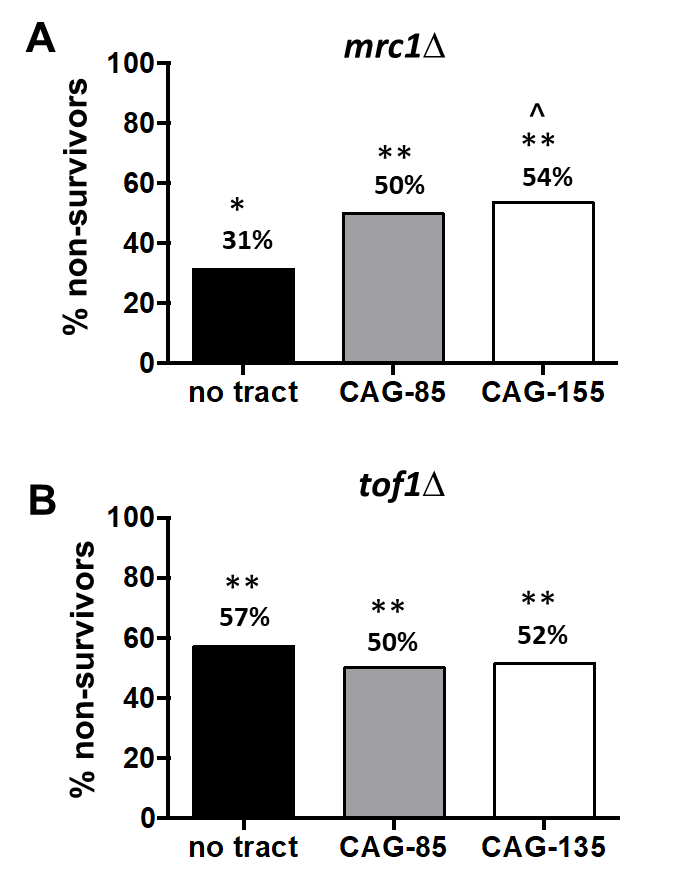


**Figure S3. Non-survivors in the absence of Tof1 or Mrc1.**

Percentage of non-survivors (area <0.016 mm^2^ at 30 hours) that arrested within the first few divisions in strains containing zero (black), 85 (gray) or 135-155 (white) CAG repeats. Significance compared to the WT value for the same tract length (*) or to the no tract value of the same strain (^) was determined by a Fisher’s exact test, * or ^ P < 0.05; ** or ^^ P < 0.01. Exact p values and numbers of colonies analyzed are in Table S3.

**Supplemental Tables**

Table S1. Supplementary Fragility Data

| **Genotype** | **Strain Background** | **CAG Repeat Length** | **# of Assays** | **Mean FOA^R^ x 10^-6^** | **Fold over WT same tract length** | **SEM** | ***P*-value compared to WT same tract length** | ***P*-value compared to no tract^** |
| --- | --- | --- | --- | --- | --- | --- | --- | --- |
| WT | BY4705 | 0 | 6 | 2.86 |  | 0.582 |  |  |
| WT | W303 | 0 | 7 | 2.32 |  | 0.393 |  |  |
| *tof1* | BY4705 | 0 | 3 | 4.12 | 1.4 | 1.06 | 0.2902 |  |
| *tof1* | W303 | 0 | 3 | 4.90 | 2.1 | 0.74 | 0.0077 |  |
| *mrc1* | BY4705 | 0 | 3 | 13.9 | 4.8 | 2.99 | 0.0013 |  |
| *mrc1* | W303 | 0 | 5 | 11.7 | 5.0 | 2.28 | 0.0007 |  |
| *mrc1AQ* | W303 | 0 | 4 | 7.28 | 3.1 | 0.803 | **<**1 x 10^-4^ |  |
| *mrc1-1* | W303 | 0 | 3 | 7.46 | 3.2 | 1.90 | 0.0041 |  |
| *rad53-21* | W303 | 0 | 5 | 6.71 | 2.9 | 0.868 | **<**1 x 10^-4^ |  |
| WT | BY4705 | 70 | 3 | 9.27 |  | 0.567 |  | 0.0002 |
| *tof1* | BY4705 | 70 | 4 | 6.64 | 0.67 | 0.599 | 0.0274 | 0.0767 |
| *mrc1* | BY4705 | 70 | 3 | 101 | 11 | 33.2 | 0.0506 | 0.0592 |
| WT | W303 | 85 | 7 | 5.15 |  | 0.554 |  | 0.0013 |
| *tof1* | W303 | 85 | 3 | 12.7 | 2.4 | 1.44 | **<**1 x 10^-4^ | 0.0017 |
| *mrc1* | W303 | 85 | 8 | 87.8 | 17 | 13.6 | **<**1 x 10^-4^ | 0.0012 |
| *mrc1AQ* | W303 | 85 | 4 | 7.94 | 1.5 | 0.560 | 0.0096 | 0.5240 |
| *mrc1-1* | W303 | 85 | 4 | 13.4 | 2.6 | 1.34 | **<**1 x 10^-4^ | 0.0446 |
| *rad53-21* | W303 | 85 | 5 | 19.4 | 3.7 | 1.46 | **<**1 x 10^-4^ | <1 x 10^-4^ |
| *tof1* | BY4705 | 110 | 3 | 73.3 | 3.7* | 17.5 | 0.03790* | 0.0002 |
| WT | BY4705 | 155 | 3 | 28.0 |  | 2.31 |  | <1 x 10^-4^ |
| WT | W303 | 155 | 5 | 9.92 |  | 2.15 |  | 0.0021 |
| *tof1* | BY4705 | 155 | 7 | 122 | 4.4 | 14.9 | 0.0040 | 0.0011 |
| *tof1* | W303 | 135 | 4 | 117 | 12 | 27.8 | **<**1 x 10^-4^ | 0.0026 |
| *mrc1* | BY4705 | 155 | 3 | 122 | 4.4 | 17.1 | 0.0055 | 0.0034 |
| *mrc1* | W303 | 155 | 8 | 146 | 15 | 17.8 | **<**1 x 10^-4^ | 0.0001 |
| *mrc1AQ* | W303 | 145 | 5 | 17.9 | 1.8 | 4.47 | 0.1464 | 0.0766 |
| *mrc1-1* | W303 | 155 | 3 | 36.2 | 3.6 | 3.93 | 0.0006 | 0.0028 |
| *rad53-21* | W303 | 135 | 7 | 34.3 | 3.5 | 3.14 | 0.0002 | <1 x 10^-4^ |

^of the same genotype. *compared to predicted WT BY4705 value of 19.7 based on a best fit line

Table S2. Supplementary Instability Data

|  | | **Contractions** | | | **Expansions** | | |  |
| --- | --- | --- | --- | --- | --- | --- | --- | --- |
| **Genotype** | **CAG Repeat Length** | **number** | **% (fold over WT)** | **p-value compared to WT same tract length** | **number** | **% (fold over WT)** | ***P*-value compared to WT same tract length** | **Total # reactions** |
| WT | 85 | 11 | 6.0 |  | 2 | 1.1 |  | 184 |
| *tof1*∆ | 85 | 54 | 26 (4.4) | **<**1 x 10^-4^ | 3 | 1.4 (1.3) | 1 | 207 |
| *mrc1*∆ | 85 | 80 | 24 (4.0) | **<**1 x 10^-4^ | 20 | 6.0 (5.5) | 0.0062 | 335 |
| *mrc1AQ* | 85 | 35 | 11 (1.8) | 0.0766 | 9 | 2.8 (2.5) | 0.3425 | 319 |
| *mrc1-1* | 85 | 16 | 10 (1.7) | 0.1626 | 9 | 5.8 (5.3) | 0.0268 | 156 |
| *rad53-21* | 85 | 32 | 21 (3.4) | **<**1 x 10^-4^ | 7 | 4.5 (4.1) | 0.0858 | 156 |
| WT | 135 | 28 | 18 |  | 2 | 1.3 |  | 155 |
| *tof1*∆ | 135 | 135 | 69 (3.8) | **<**1 x 10^-4^ | 3 | 1.5 (1.2) | 1 | 197 |
| *mrc1*∆ | 155 | 214 | 67 (3.7) | **<**1 x 10^-4^ | 22 | 7.0 (5.3) | 0.007 | 319 |
| *mrc1AQ* | 145 | 129 | 43 (2.4) | **<**1 x 10^-4^ | 9 | 3.0 (2.3) | 0.3457 | 299 |
| *mrc1-1* | 155 | 47 | 30 (1.7) | 0.0167 | 2 | 1.3 (1.0) | 1 | 156 |
| *rad53-21* | 135 | 58 | 37 (2.1) | **<**1 x 10^-4^ | 3 | 1.9 (1.5) | 1 | 155 |

Tables S3. Microcolony Data and Statistical Analysis

|  | **area mean, mm^2^ (total number of microcolonies analyzed)** | | | ***P*-value, Fisher’s LSD Test** | |
| --- | --- | --- | --- | --- | --- |
|  | no tract | medium tract | long tract | medium tract  vs no tract | long tract  vs no tract |
| **strain** | **MICROCOLONY ALL** | | | | |
| **WT** | 0.3437 (20) | 0.1969 (20) | 0.2224 (18) | **<**1 x 10^-4^ | **<**1 x 10^-4^ |
| ***mrc1-AQ*** | 0.1678 (15) | 0.0596 (15) | 0.07196 (15) | **<**1 x 10^-4^ | **<**1 x 10^-4^ |
| ***rad53-21*** | 0.03436 (82) | 0.06971 (41) | 0.03827 (40) | **<**1 x 10^-4^ | 0.656 |
|  | **MICROCOLONY SURVIVORS^** | | | | |
| **WT** | 0.3617 (19) | 0.2072 (19) | 0.2355 (17) | **<**1 x 10^-4^ | **<**1 x 10^-4^ |
| ***mrc1-AQ*** | 0.1792 (14) | 0.0596 (15) | 0.07599 (14) | **<**1 x 10^-4^ | **<**1 x 10^-4^ |
| ***rad53-21*** | 0.08693 (32) | 0.08881 (32) | 0.06953 (22) | 0.8124 | 0.0496 |

^survivors defined as microcolonies with area ≥0.016 mm^2^ at 30 hours

| **NON-SURVIVORS^** | **number of nonsurvivors**  **(total # of microcolonies analyzed)** | | | ***P*-value, Fisher’s Exact Test** | | |
| --- | --- | --- | --- | --- | --- | --- |
| **strain** | no tract | medium tract | long tract | medium tract vs no tract | long tract vs no tract | long tract vs medium tract |
| **WT** | 1 (20) | 1 (20) | 1 (18) | 1 | 1 | 1 |
| ***mrc1-AQ*** | 2 (32) | 3 (30) | 4 (32) | 0.666 | 0.672 | 1 |
| ***rad53-21*** | 50 (82) | 9 (41) | 18 (40) | 0.000049 | 0.1209 | 0.0352 |
| ***mrc1*∆** | 16 (51) | 22 (44) | 29 (54) | 0.0926 | 0.0297 | 0.839 |
| ***tof1*∆** | 13 (28) | 19 (38) | 17 (33) | 0.807 | 0.7988 | 1 |

^non-survivors defined as microcolonies with area <0.016 mm^2^ at 30 hours

| **NON-SURVIVORS^** | ***P*-value, Fisher’s Exact Test** | | |
| --- | --- | --- | --- |
| Strains being compared | no tract | medium tract | long tract |
| ***mrc1-AQ* vs. WT** | 1 | 0.64 | 0.64 |
| ***rad53-21* vs. WT** | 6.1 x 10^-6^ | 0.14 | 0.0027 |
| ***rad53-21* vs. *mrc1-AQ*** | 4.29 x 10^-8^ | 0.22 | 4.2 x 10^-3^ |
| ***mrc1*∆ vs. WT** | 0.0028 | 5.0 x 10^-4^ | 2.4 x 10^-4^ |
| ***tof1*∆ vs. WT** | 0.0029 | 4.6 x 10^-4^ | 0.0016 |

^non-survivors defined as microcolonies with area <0.016 mm^2^ at 30 hours. Comparisons were made between the indicated strains for the same tract length.

Table S4. Strain Table

| **Strain Number** | **Strain Name** | **Strain Background** | **Genotype** | **Reference** |
| --- | --- | --- | --- | --- |
| CFY765 | WT CAG-0 | BY4705 | *Mat α, ade2Δ::hisG, his3Δ200, leu2Δ0, lys2Δ0, met15Δ0, trp1Δ63, ura3Δ0, can^R^;* YAC: *LEU2, URA3,* CAG-0 | (Sundararajan et al. 2009) |
| CFY766 | WT CAG-70 | BY4705 | *Mat α, ade2Δ::hisG, his3Δ200, leu2Δ0, lys2Δ0, met15Δ0, trp1Δ63, ura3Δ0, can^R^;* YAC: *LEU2, URA3,* CAG-70 | (Sundararajan et al. 2009) |
| CFY767 | WT CAG-155 | BY4705 | *Mat α, ade2Δ::hisG, his3Δ200, leu2Δ0, lys2Δ0, met15Δ0, trp1Δ63, ura3Δ0, can^R^;* YAC: *LEU2, URA3,* CAG-155 | (Sundararajan et al. 2009) |
| CFY813, CFY814 | WT Y80 CAG-0 | W303, Y300 | *Mat a, ade2-1, his3-11,15, leu2-3,112, trp1-1, ura3-1, can1-100;* YAC: *LEU2, URA3,* CAG-0 | Lahiri et al., 2004 |
| CFY859, CFY860 | WT Y80 CAG-85 | W303, Y300 | *Mat a, ade2-1, his3-11,15, leu2-3,112, trp1-1, ura3-1, can1-100;* YAC: *LEU2, URA3,* CAG-85 | Lahiri et al., 2004 |
| CFY874, CFY875 | WT CAG-135 | W303, Y300 | *Mat a, ade2-1, his3-11,15, leu2-3,112, trp1-1, ura3-1, can1-100;* YAC: *LEU2, URA3,* CAG-135 | Lahiri et al., 2004 |
| CFY1685, CFY1763 | WT CAG-155 | W303 | *Mat a, ade2-1, his3-11,15, leu2-3,112, trp1-1, ura3-1, can1-100;* YAC: *LEU2, URA3,* CAG-155 | This study |
| CFY2610 | *tof1::HIS3* CAG-0 | BY4705 | *Mat α, ade2Δ::hisG, his3Δ200, leu2Δ0, lys2Δ0, met15Δ0, trp1Δ63, ura3Δ0, tof1::HIS3;* YAC: *LEU2, URA3,* CAG-0 | This study |
| CFY2615 | *tof1::HIS3* CAG-70 | BY4705 | *Mat α, ade2Δ::hisG, his3Δ200, leu2Δ0, lys2Δ0, met15Δ0, trp1Δ63, ura3Δ0, tof1::HIS3;* YAC: *LEU2, URA3,* CAG-70 | This study |
| CFY2616 | *tof1::KANMX* CAG-85 | BY4705 | *Mat α, ade2Δ::hisG, his3Δ200, leu2Δ0, lys2Δ0, met15Δ0, trp1Δ63, ura3Δ0, tof1::KANMX;* YAC: *LEU2, URA3,* CAG-85 | This study |
| CFY2614 | *tof1::HIS3* CAG-110 | BY7405 | *Mat α, ade2Δ::hisG, his3Δ200, leu2Δ0, lys2Δ0, met15Δ0, trp1Δ63, ura3Δ0, tof1::HIS3;* YAC: *LEU2, URA3,* CAG-110 | This study |
| CFY2617, CFY2590 | *tof1::KANMX* CAG-155 | BY4705 | *Mat α, ade2Δ::hisG, his3Δ200, leu2Δ0, lys2Δ0, met15Δ0, trp1Δ63, ura3Δ0, tof1::KANMX;* YAC: *LEU2, URA3,* CAG-155 | This study |
| CFY4282, 4283 | *tof1::KANMX6* CAG-0 | W303, Y300 | *Mat a, ade2-1, his3-11,15, leu2-3,112, trp1-1, ura3-1, can1-100, tof1::KANMX6;* YAC: *LEU2, URA3,* CAG-0 | This study |
| CFY1213 | *tof1*::*HIS3MX6* CAG-85 | W303 | *Mat a, ade2-1, his3-11,15, leu2-3,112, trp1-1, ura3-1, can1-100, tof1::HIS3MX6;* YAC: *LEU2, URA3,* CAG-85 | This study |
| CFY1214 | *tof1*::*HIS3MX6* CAG-135 | W303 | *Mat a, ade2-1, his3-11,15, leu2-3,112, trp1-1, ura3-1, can1-100 tof1::HIS3MX6;* YAC: *LEU2, URA3,* CAG-135 | This study |
| CFY2287, CFY2288 | *mrc1::HIS3* CAG-0 | BY4705 | *Mat α, ade2Δ::hisG, his3Δ200, leu2Δ0, lys2Δ0, met15Δ0, trp1Δ63*, *ura3Δ0, mrc1::HIS3;* YAC: *LEU2, URA3,* CAG-0 | This study |
| CFY2287, CFY2288 | *mrc1::HIS3* CAG-0 | BY4705 | *Mat α, ade2Δ::hisG, his3Δ200, leu2Δ0, lys2Δ0, met15Δ0, trp1Δ63*, *ura3Δ0, mrc1::HIS3;* YAC: *LEU2, URA3,* CAG-0 | This study |
| CFY2591 | *mrc1*::*HIS3* CAG-155 | BY4705 | *Mat α, ade2Δ::hisG, his3Δ200, leu2Δ0, lys2Δ0, met15Δ0, trp1Δ63, ura3Δ0, mrc1::HIS3;* YAC: *LEU2, URA3,* CAG-155 | This study |
| CFY1633, CFY1634 | *mrc1::KANMX4* CAG-0 | W303 | *Mat α, ade2-1, his3-11,15, leu2-3,112, trp1-1, ura3-1, can^R^, hom3-10, mrc1::KANMX4;* YAC: *LEU2, URA3,* CAG-0 | This study |
| CFY1635, CFY1636 | *mrc1*::*KANMX4* CAG-85 | W303 | *Mat α, ade2-1, his3-11,15, leu2-3,112, trp1-1, ura3-1, can^R^, hom3-10, mrc1::KANMX4;* YAC: *LEU2, URA3,* CAG-85 | This study |
| CFY1637, CFY1638 | *mrc1*::*KANMX4* CAG-155 | W303 | *Mat α, ade2-1, his3-11,15, leu2-3,112, trp1-1, ura3-1, can^R^, hom3-10, mrc1::KANMX4;* YAC: *LEU2, URA3, CAG-0;* YAC: *LEU2, URA3,* CAG-155 | This study |
| CFY1104, CFY1105 | *HIS::mrc1AQ*MYC13 CAG-0 | W303 | *Mat a, ade2-1, his3-11,15, leu2-3,112, trp1-1, ura3-1, can1-100 HIS::mrc1AQMYC13;* YAC: *LEU2, URA3,* CAG-0 | (Osborn 2003); this study |
| CFY1195, CFY1196 | *HIS::mrc1AQ*MYC13 CAG-85 | W303 | *Mat a, ade2-1, his3-11,15, leu2-3,112, trp1-1, ura3-1, can1-100 HIS::mrc1AQMYC13;* YAC: *LEU2, URA3,* CAG-85 | (Osborn 2003); this study |
| CFY1107 | *HIS::mrc1AQ*MYC13 CAG-145 | W303 | *Mat a, ade2-1, his3-11,15, leu2-3,112, trp1-1, ura3-1, can1-100, HIS::mrc1AQMYC13;* YAC: *LEU2, URA3,* CAG-145 | (Osborn 2003); this study |
| CFY947, CFY948, CFY949 | *mrc1-1* CAG-0 | W303, Y300 | *Mat a, ade2-1, his3-11,15, leu2-3,112, trp1-1, ura3-1, can1-100, mrc1-1;* YAC: *LEU2, URA3,* CAG-0 | (Freudenreich and Lahiri, 2004) |
| CFY950, CFY951, CFY952 | *mrc1-1* CAG-85 | W303, Y300 | *Mat a, ade2-1, his3-11,15, leu2-3,112, trp1-1, ura3-1, can1-100, mrc1-1;* YAC: *LEU2, URA3,* CAG-85 | (Freudenreich and Lahiri, 2004) |
| CFY953, CFY954, CFY955 | *mrc1-1* CAG-155 | W303, Y300 | *Mat a, ade2-1, his3-11,15, leu2-3,112, trp1-1, ura3-1, can1-100, mrc1-1;* YAC: *LEU2, URA3,* CAG-155 | (Freudenreich and Lahiri, 2004) |
| CFY815, CFY816 | *rad53-21* CAG-0 | W303, Y300 | *Mat a, ade2-1, his3-11, 115, leu2-3,112, trp1-1, ura3-1, can1-100, rad53-21;* YAC: *LEU2, URA3,* CAG-0 | (Lahiri et al. 2004) |
| CFY861, CFY862 | *rad53-21* CAG-85 | W303, Y300 | *Mat a, ade2-1, his3-11, 115, leu2-3,112, trp1-1, ura3-1, can1-100, rad53-21;* YAC: *LEU2, URA3,* CAG-85 | (Lahiri et al. 2004) |
| CFY876, CFY877 | *rad53-21* CAG-135 | W303, Y300 | *Mat a, ade2-1, his3-11,15, leu2-3,112, trp1-1, ura3-1, can1-100, rad53-21;* YAC: *LEU2, URA3,* CAG-135 | (Lahiri et al. 2004) |
| CFY4618 | WT pYES2_CAG-70 | W303 | *Mat a, ade2-1, his3-11,15, leu2-3,112, trp1-1, ura3-1, can1-100;* pYES2_CAG-70 | This study |
| CFY4619 | *tof1::NATMX* pYES2_CAG-70 | W303 | *Mat a, ADE2, his3-11,15, leu2-3,112, trp1-1, ura3-1, can^R^met15D0*  *tof1::NATMX* pYES2_CAG-70 | This study |
| CFY4620 | WT pYES2_CAG-130 | W303 | *Mat a, ade2-1, his3-11,15, leu2-3,112, trp1-1, ura3-1, can1-100;* pYES2_CAG-130 | This study |
| CFY4621 | *tof1::NATMX* pYES2_CAG-130 | W303 | *Mat a, ADE2, his3-11,15, leu2-3,112, trp1-1, ura3-1, can^R^met15D0*  *tof1::NATMX;*  pYES2_CAG-130 | This study |
| CFY4724 | *mrc1::KANMX4*  pYES2_CAG-70 | W303 | *Mat α, ade2-1, his3-11,15, leu2-3,112, trp1-1, ura3-1, can^R^, hom3-10, mrc1::KANMX4;* pYES2_CAG-70 | This study |
| CFY4725 | *mrc1::KANMX4*  pYES2_CAG-130 | W303 | *Mat α, ade2-1, his3-11,15, leu2-3,112, trp1-1, ura3-1, can^R^, hom3-10, mrc1::KANMX4;* pYES2_CAG-130 | This study |
